# A systematic framework for functional connectivity measures

**DOI:** 10.3389/fnins.2014.00405

**Published:** 2014-12-09

**Authors:** Huifang E. Wang, Christian G. Bénar, Pascale P. Quilichini, Karl J. Friston, Viktor K. Jirsa, Christophe Bernard

**Affiliations:** ^1^Institut de Neurosciences des Systèmes, Aix Marseille UniversitéMarseille, France; ^2^Institut national de la santé et de la recherche médicale, UMR_S 1106Marseille, France; ^3^The Wellcome Trust Centre for Neuroimaging, University College LondonLondon, UK

**Keywords:** functional connectivity, evaluation framework, neural mass models, fMRI, Granger causality

## Abstract

Various methods have been proposed to characterize the functional connectivity between nodes in a network measured with different modalities (electrophysiology, functional magnetic resonance imaging etc.). Since different measures of functional connectivity yield different results for the same dataset, it is important to assess when and how they can be used. In this work, we provide a systematic framework for evaluating the performance of a large range of functional connectivity measures—based upon a comprehensive portfolio of models generating measurable responses. Specifically, we benchmarked 42 methods using 10,000 simulated datasets from 5 different types of generative models with different connectivity structures. Since all functional connectivity methods require the setting of some parameters (window size and number, model order etc.), we first optimized these parameters using performance criteria based upon (threshold free) ROC analysis. We then evaluated the performance of the methods on data simulated with different types of models. Finally, we assessed the performance of the methods against different levels of signal-to-noise ratios and network configurations. A MATLAB toolbox is provided to perform such analyses using other methods and simulated datasets.

## Introduction

Information exchange occurs at multiple scales in the brain, from protein-protein interactions in cells to communication among cortical regions. Information can be coded in different manners (e.g., chemical, electrical) and measured with different modalities, including magneto- and electro-encephalography (MEG, EEG), and functional Magnetic Resonance Imaging (fMRI). These techniques allow one to characterize the architecture generating time series in terms of functional coupling. There are two approaches to functional coupling; namely, the assessment of functional connectivity and the estimation of effective connectivity. Functional connectivity is defined as the statistical dependence among measured time series—usually evaluated in terms of correlations or mutual information. These measures have recently been supplemented with methods like multivariate Granger causality and directed transfer functions to provide measures that are less sensitive to indirect links. In contrast, effective connectivity quantifies the directed (causal) influence of one neuronal system over another and relies upon a model of neuronal coupling (Friston, 1994; Bullmore and Sporns, 2012). Both functional and effective connectivity can be represented in terms of a graph, where the nodes of the graph represent cortical or subcortical areas and the edges correspond to (directed or undirected) connections (Bullmore and Sporns, 2012). In this technical note, we will focus on methods for assessing functional connectivity.

Different functional connectivity methods can give different results, as they are based on different underlying mathematical assumptions or measures of dependency (Smith et al., 2011). It may therefore be difficult to select the method that is best suited for identifying the appropriate graph or quantifying functional connection strengths. In this context, simulated data plays an essential role in evaluating competing methods against a “ground truth.” Analyzing data generated by a known architecture enables one to optimize various analysis parameters and assess dependencies on the generative process and robustness to analysis assumptions.

Various methods for assessing functional connectivity have already been reviewed (Pereda et al., 2005) and tested using fMRI simulated signals (Smith et al., 2011), mathematical models (Ansari-Asl et al., 2006; Wendling and Ansari-Asl, 2009) and neural mass models (David et al., 2004). These methods include correlation (Rodgers and Nicewander, 1988), coherence (Lopes da Silva et al., 1989), mutual information (Grassberger et al., 1991), transfer entropy (Schreiber, 2000), directed coherence, Granger causality (Granger, 1969), generalized synchronization (Quiroga et al., 2002) and Bayes net methods (Ramsey et al., 2006) etc. Methods for evaluating effective connectivity include Dynamic causal modeling (DCM) (Friston et al., 2003) and structural equation modeling (Mclntosh and Gonzalez_Lima, 1994). However, no evaluation framework exists to systematically compare the results of different methods for various types of datasets. We introduce here such evaluation framework, and we show its usefulness performing a systematic analysis of 42 methods using multiple datasets with different structures. Note that our goal here is not determine why a given method succeeds or fails in a specific condition. Our goal is to provide neuroscientists with a useful tool to make informed choices about the method one wishes to use according to the type of data. The evaluation framework uses different connectivity architectures and biologically realistic models of various levels of complexity and time scales. We included 42 methods to span the domain of application of functional connectivity schemes. In this paper, we focus on the taxonomy of functional connectivity schemes and the generative models used to create simulated data, with a special focus on the sensitivity of different schemes to analysis parameters, the architecture of the generative models and different types and levels of noise.

As an example of the proposed systematic evaluation framework, we benchmarked 42 methods against more than 10^4^ datasets generated under five classes of simulated models. We assessed the performance and robustness of all methods by asking; which methods can infer the right connectivity architecture and under what conditions? What are the appropriate parameters for the methods to recover the underlying architecture correctly? Can functional connectivity be evaluated quantitatively for any given architecture? What is the accuracy of these methods under variations of the underlying architecture? And, finally, how robust are these methods against different levels of observation noise? We used generic and simple criteria to assess performance—so that the relative performance over different schemes could be compared. Our interest here was not in optimizing any particular scheme with bespoke performance measures. We wanted to establish complementary domains of sensitivity that could be used in subsequent work to exploit the plurality of functional connectivity measures considered in this paper.

## Methods

**Figure 1:**
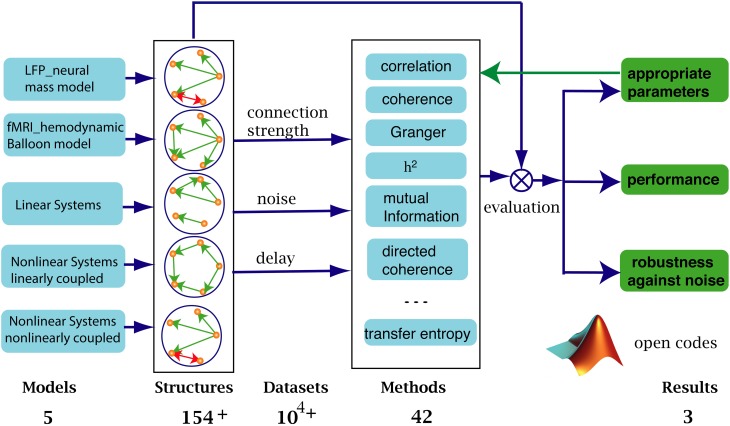
**We benchmarked 42 methods against more than 10^4^ simulated datasets from 5 types of models with different connectivity structures. Connection strengths, noise levels and time delays were varied to test the robustness of the methods**. The same procedure was used to identify the proper range of parameters specifically used in the different methods. The performance of a given method was evaluated by comparing the computed graph to the ground truth structure.

Figure 1 shows how our systematic evaluation framework works. We simulated five types of data (first column) to cover the dynamic characteristics (time scales, complexity, and multi-scale properties) of signals obtained from EEG and fMRI modalities, including linear and non-linear systems with linear or non-linear coupling. In terms of connectivity architectures, we considered at least 154 underlying structures (all possible topologies for five nodes and five edges), with varying connection strengths, noise levels and time delays (third column). We applied 42 connectivity analysis methods (fourth column) to the ensuing datasets and compared the resulting connectivity with the ground-truth structures.

Using this setup, we (1) identified the valid (optimal) parameter range for each method. (2) Using this parameter range, we evaluated the relative performance of each method and, (3) finally, we characterized the accuracy of each method in the face of different levels of system noise (innovations or random fluctuations producing simulated neuronal signals) and four types of observation noise (random fluctuations added to signal to simulate data).

In what follows, we consider each of these components in detail. An open source MATLAB code is available to test other methods and identify optimal analysis parameters. Our numerical simulations were performed using The Virtual Brain (TVB) high performance computing platform using 240 Intel Xeon E5–2670 based computing cores^1^.

### Generative models

We started with two types of generative model, simulating EEG and fMRI signals in a biologically realistic way. We used two generative models: a convolution-based neural mass model for EEG (Moran et al., 2013) and a non-linear haemodynamic model for fMRI (Friston et al., 2003). Since the various functional connectivity methods are known to have specific sensitivities to nonlinearities in the data, we also analyzed three more abstract (mathematical) categories of generative models for each node (region or source): linear systems, non-linear systems with linear coupling and non-linear systems with non-linear coupling. Their specification can be found in Supplementary Section 1.

We considered synaptic convolution-based neural mass models (NMM) in order to test the methods at the millisecond timescale, which is typical of EEG signals. These models (Moran et al., 2013) consider cortical columns with three subpopulations: spiny stellate cells in granular layer IV, pyramidal cells and inhibitory interneurons in extra granular layers (II and III, V and VI), as shown in Supplementary Figure 1. The neuronal states of these generative models include currents and membrane potentials. A connection between two columns i and j corresponds to a link between the pyramidal subpopulation of column i and the spiny stellate subpopulation of column j. We also added variable delays between columns, system noise on spiny stellate cells and observation noise on the signals.

We used the standard generative model used in DCM to generate simulated BOLD signals. The neuronal signals were generated using linear differential equations with one neuronal state per node (Friston et al., 2003; Stephan et al., 2007) as shown in Supplementary Figure 2. This neural activity is transformed into a BOLD response using a non-linear haemodynamic model (incorporating the empirically validated balloon model). The associated haemodynamic response function effectively acts as a non-linear convolution operator to remove high-frequency fluctuations in neuronal signals. This means simulated BOLD time series have a slower timescale as compared to EEG signals. We used the BOLD signal to analyze the underlying connectivity between the neuronal states of nodes i and j. We also added system noise to the neuronal state and observation noise to the BOLD signals.

In addition to these biophysically plausible electrophysiological and haemodynamic models, we considered three more abstract (mathematical) generative models: we started with coupled linear systems. We then considered non-linear systems based on Rössler attractors (Rössler, 1979), where the nodes were coupled linearly. We also simulated time series with non-linear systems based on Hénon maps (Hénon, 1976), with non-linear coupling among nodes. The range of time scales and complexity present in time series commonly acquired with invasive (stereotactic EEG) and non-invasive (EEG, MEG, fMRI) methods is large. We chose to include neurally motivated models [NMM from Jansen-Rit (Jansen and Rit, 1995), fMRI from Ballon-Windkessel (Friston et al., 2003)], and well-investigated dynamic models with no direct reference to neural systems (Rössler, Hénon, linear stochastic system). We chose these models because they cover different systemic properties, i.e., linear and non-linear systems with linear and non-linear coupling. These properties are generally not known in real datasets. Hence our goal was to cover all possibilities. Another motivation was to facilitate comparison with previously published work on the assessment of analysis methods (Quiroga et al., 2002; Ansari-Asl et al., 2006; Wendling and Ansari-Asl, 2009).

**Figure 2:**
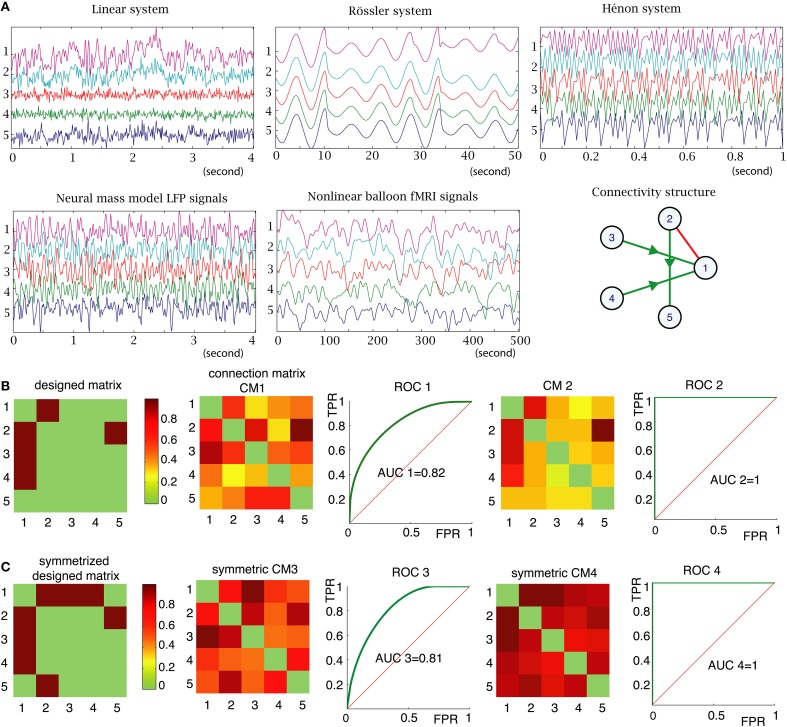
**(A)** Examples of signals generated by the 5 types of models in the same 5 nodes structure (right part of the middle panel). The y-axis units represent the node number and the x-axis units are time-series in seconds with different sampling rates and time scales. **(B)** Left: “ground truth” connectivity matrix, which is another representation of the connectivity structure shown in **(A)**. The color code corresponds to the strength of the connections between nodes (set to 1 for all connections in this example). Connection matrix (CM1) was obtained with PDC method. ROC 1 is the receiver operating characteristic (ROC) curve, which displays true positive rate as a function of false positive rate, for the PDC method, measured for all connection strength values (strength threshold). The AUC is the area under the curve. If AUC is 1 (as for CM2 and ROC2), it means that the method is able to find the “ground truth,” if given the correct threshold. **(C)** For the undirected methods, such as BCorrU, the computed connection matrices are symmetric, thus we took the symmetrized designed matrix as a ground truth for calculating their ROC and AUC.

Figure 2A illustrates five types of simulated time series with different signal dynamics and different time resolutions for a given connectivity structure. Because the different types of signals have different sample frequencies, we used the number of time points as a measure of time to define analysis parameters, such as the size of sliding window, the length of the time series and time delays.

### Functional connectivity measures

We chose 42 functional connectivity measures from 7 families: correlation, h^2^, mutual information, coherence, Granger, transfer entropy and 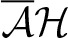 (MVAR-frequency domain based techniques, see below for details). The mathematical form of these measures of functional connectivity can be found in Supplementary Table 1 of Supplementary Section 2. We selected these methods because they have low computational cost and do not require prior knowledge of the underlying generative model. We now present the different methods for each family of measures.

#### Correlation family

The correlation (Corr) family of measures is based on the Pearson correlation coefficient (Rodgers and Nicewander, 1988) and considers time delays. Four methods can be distinguished: BCorrD, PCorrD, BCorrU, and PCorrU. The prefix B indicates bivariate methods using pair-wise calculations. The prefix P indicates partial results from the bivariate results using matrix inversion (the connectivity is evaluated between any two nodes while considering the influence of the other nodes). If the bivariate matrix is sparse, we estimated a sparse inverse covariance matrix using a Lasso (L1) penalty (http://www.cs.ubc.ca/~schmidtm/Software/L1precision.html). The suffixes U and D indicate undirected and directed connectivity, respectively.

For example, BCorrD is the bivariate correlation method for measuring directed connectivity. The direction i → j is determined if past of i has a stronger correlation with current j than past of j has with current i. BCorrU is the bivariate correlation method for measuring undirected connectivity by selecting the largest value between i → j and j → i. PCorrD (PCorrU) is the partial result from BCorrD (BCorrU). Partial methods suppress the functional connectivity induced by indirect effective connectivity as well as dependencies between two nodes that receive input from a common source.

#### h^2^ family

The h2 family calculates non-linear correlation coefficients based on the fitting of a non-linear curve from the piece-wise linear approximation between two time series (Lopes da Silva et al., 1989; Wendling et al., 2001; Ansari-Asl et al., 2006). As for the Corr family, Bh2D is the bivariate h^2^ method for directed connectivity and Ph2D is its partial equivalent. Bh2U is the bivariate h^2^ method for undirected connectivity and Ph2U is its partial version.

#### Mutual information in time domain

Mutual information estimates the shared information between two time series based on Shannon entropy. For computational expediency, we only considered the mutual information in the time domain (MIT) (Grassberger et al., 1991; Paluš et al., 2001; Quiroga et al., 2002). BMITD1 measures functional connectivity by comparing the individual histograms of two signals to the joint histograms—taking into account time delays. BMITD2 reduces the (discretization) bias of the estimated entropy of two series. BMITU infers the undirected connectivity, and PMITD1, PMITD2, and PMITU are the partial versions. In theory, mutual information can measure both linear and non-linear dependencies.

#### Granger causality family

The Granger causality (GC) family calculates the directed connectivity (j→ i) based on the notion that information in the past of j helps to predict the future of i with greater accuracy than by only considering the past of i itself (Granger, 1969). We used two methods from “Granger causal connectivity analysis (GCCA)” Matlab toolbox (Seth, 2010), GC and CondGC. These methods are based on multivariate autoregressive models (MVAR):

(1) $$\text{X}\left(\text{t}\right)=\sum_{\text{r }=\;1}^{\text{p}}{\text{A}}_{\text{r}}\text{X}\left(\text{t}-\text{r}\right)+\Xi \left(\text{t}\right)$$

With state vector X (t) = x_1_ (t), ···, x_n_ (t)]′_n × 1_ and prediction errors Ξ (t) = [ξ_1_ (t), ···, ξ_n_ (t)]′_n × 1_ for n nodes. p is the model order (maximum number of lagged observations included in the model) for the MVAR and r is the time delay. Omitting x_j_ from X, we obtain a second group of MVAR with another prediction errors. GC measures the connection strength j → i by comparing residuals (based on the above MVAR model) without and with x_j_. CondGC, for conditional Granger causality [also called Partial G-Causality in (Seth, 2010)], considers the influence from exogenous and/or latent variables on measured responses. This information may be reflected in the correlations among the residuals of an MVAR model of observed responses. As with the other families, we also used PGC as the partial method.

#### Transfer entropy (TE)

Transfer entropy (Schreiber, 2000) measures the directed connectivity (j→i) if the joint information of the past j, i can reduce the degree of uncertainty in the future values of x_i_ compared with the degree of uncertainty of x_i_ only from the past of x_i_. Granger causality and transfer entropy are equivalent for Gaussian variables (Barnett et al., 2009). However, transfer entropy requires much less computational time than Granger causality for high model orders and larger numbers of nodes. We thus used BTED to compute the directed connectivity for Gaussian variables (Chicharro, 2011) taking into account time delays. We also considered PTED for partial BTED, BTEU for the undirected connectivity and PTEU for the partial undirected connectivity methods.

#### Coherence

Coherence (Coh) calculates the undirected connectivity based on the cross-correlation in the frequency domain (Hinich and Clay, 1968; Grinsted et al., 2004). We tested bivariate coherence with two different types of transforms: BCohF for Fourier transforms and BCohW for Wavelet transforms. We also tested the partial versions PCohF and PCohW.

#### 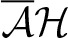 family

The 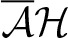 family calculates the connectivity based on the coefficients of the MVAR in the frequency domain. From MVAR (1), we can get $\Xi \left(\text{t}\right)=\text{X}(\text{t})-\sum_{\text{r}=\text{1}}^{\text{p}}{\text{A}}_{\text{r}}\text{X}\left(\text{t}-\text{r}\right)=\sum_{\text{r}=\text{0}}^{\text{p}}{\bar{\text{A}}}_{\text{r}}\text{X}\left(\text{t}-\text{r}\right)$, with A_r_ = I − A_r_. Seventeen methods have been introduced based on the transforms of A_r_ (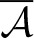
(*f*)) and its inversion 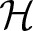 = 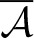
^−1^, Thus we give this family the name 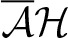.

Af presents the results from 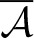
(*f*), and PDC (partial directed coherence) is the normalized Af method for ranking the relative interaction strengths with respect to a given signal source, i.e., the influence of the past x_j_ over current x_i_ compared to the past x_j_ over other signals (Baccalá and Sameshima, 2001). Partial directed coherence factor (PDCF) in (Baccalá and Sameshima, 2001) considers instantaneous effects. Generalized partial directed coherence (GPDC) uses a weighting function to remedy the unbalanced residuals from PDC (Baccalá et al., 2006; Baccalá, 2007). These methods compose the 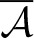 group.

Hmvar presents the results from 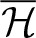
(*f*) and directed transfer function (DTF) is the normalized Hmvar (Kaminski and Blinowska, 1991; Kaminski et al., 2001). DTF calculates the directed connectivity by ranking the relative interaction strengths with respect to a given input; i.e., the influence of past x_j_ over current x_i_ compared to the influence of past other signals over current x_i_. Full frequency directed transfer function ffDTF normalizes the connectivity over the considered frequency band (Kaminski and Liang, 2005) Directed coherence DC considers the information from the residuals (Baccalá et al., 1998) DC/DTF can be regarded as the partial equivalents of PDCF/PDC. Geweke's Granger Causality (GGC) can be seen as Granger causality in the frequency domain (Geweke, 1982, 1984). These methods compose the H group.

The cross-spectral power density matrix (Smvar) can also be obtained from 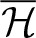
(*f*) and a covariance matrix of residuals. Ordinary coherences (COH1 and COH2) are the normalized methods of Smvar with/without considering prediction errors. PCOH1 and PCOH2 are their partial ordinary coherences. Direct directed transfer function (dDTF) combines the information from ffDTF and PCOH2 (Korzeniewska et al., 2003). This concludes our summary of the functional connectivity measures we considered.

#### The functional connectivity analysis methods

**Figure 3:**
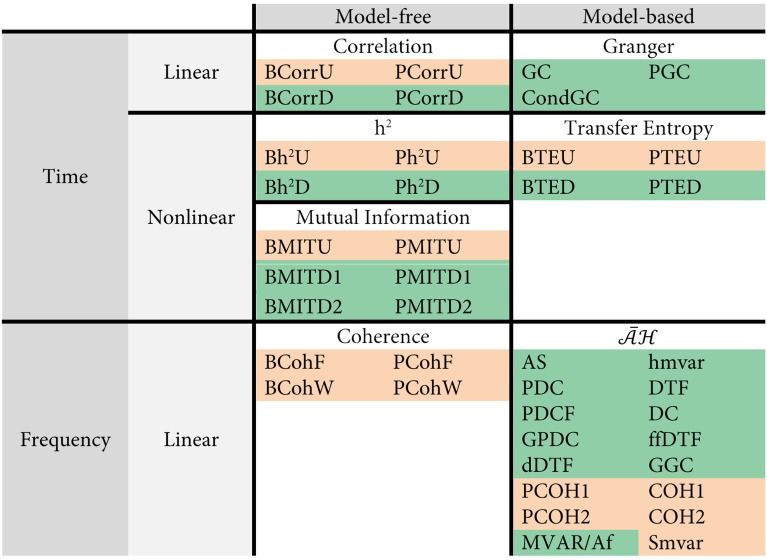
**Categorization of the 42 connectivity analysis methods used here**. The methods yielding undirected structures are highlighted in pink and the methods yielding directed structures are highlighted in green.

We first divided these 42 connectivity analysis methods into two major classes: model-free (MF) and model-based (MB) according to their dependency upon autoregressive models for estimating statistical dependencies, as shown in Figure 3. For example, correlation, h2, mutual information and coherence are model-free, and the Granger family, its equivalent transfer entropy and 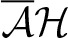 are model-based. Coherence and the 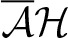 family work in the frequency domain, and the other 5 families work in the time domain. Mutual information, h2 and transfer entropy are non-linear methods, while the remaining are linear methods.

Most of 42 connectivity measures have been previously published, but some are introduced in this paper as extensions of established measures. We have thus added the BCorrD, PCorrD, Bh2D, Ph2D, BTEU, PTEU, PTED, PMITD1, and PMITD2, PGC methods in order to account for bivariate, partial, directed and undirected versions of the existing measures. The mathematical details are available for interested readers in the Supplementary Table 1 of Supplementary Section 2.

### Evaluation of the methods

For each simulated dataset, each method computes a functional connectivity matrix, which assigns a connection value between 0 and 1 between any two nodes (Figure 2B). When analyzing real datasets of unknown structure, the connection strength can be thresholded to produce a network graph. For example, in the connection matrix 2 in Figure 2B, choosing a connection threshold of 0.4 will produce a graph with many more links (those in orange color) than the ground truth structure. However, with the threshold of 0.5, the method identifies the ground truth structure. Choosing the most appropriate threshold value is an unresolved problem. However, simulated data is generated with a designed structure or graph, which we refer to as a ground truth structure. Based on these ground truth structures, we can calculate the number of false positive and false negative edges as a function of threshold. This enables us to compute receiver operating characteristic (ROC) curves (Zweig and Campbell, 1993) and evaluate the area under the curve (AUC) (Figure 2B). Note that, for the undirected methods, the computed connection matrices are symmetric, thus we took the symmetrized designed matrix as a ground truth for calculating their ROC and AUC (Figure 2C). The closer the AUC is to 1, the closer the measured graph is to the true underlying graph. In the following, if AUC= 1, we consider that the method is able to identify the underlying structure; i.e., there exists a threshold for which the method can identify the correct structure. This represents a simple universal performance criterion that can be applied to any functional connectivity measure (based upon simulated data).

For each of the 5 generative models, and for each parameter value (noise etc.), we simulated data from 154 structures (all combinations of 5 edges in a 5 node graph). We then averaged the corresponding 154 AUC results to quantify the performance of each method. If this average AUC value was close to 1, we considered that the method could recover most, if not all, 154 possible structures. We now report the results of our assessment.

## Results

### Analysis parameters

To analyze a given dataset, we used a sliding window (with a given overlap) to analyze each timeseries. In other words, the timeseries was analyzed in N windows and a connection matrix was calculated for each window. These N matrices were then averaged into a mean connection matrix. We then compared this mean connection matrix with the designed one to obtain an AUC value. Each method requires setting some parameters, which affect their performance. These parameters include the size of any sliding window, the number of windows, the length of time series analyzed, the frequency bands for methods using the frequency domain, the maximum lag for the model-free methods, and the model order for the model-based methods. We therefore first performed a parametric study to determine the valid range of values for a given method using NMM and fMRI signals from the biophysical generative models.

Furthermore, since the ability of methods to detect the presence of an edge between two nodes depends upon the strength of this link, we varied the effective connection strengths (CS) in the generative models. In order to evaluate the performance of methods for different CS values, all effective connections (edge weights) were assigned the same CS (between 0 and 1).

#### Size of sliding windows

**Figure 4:**
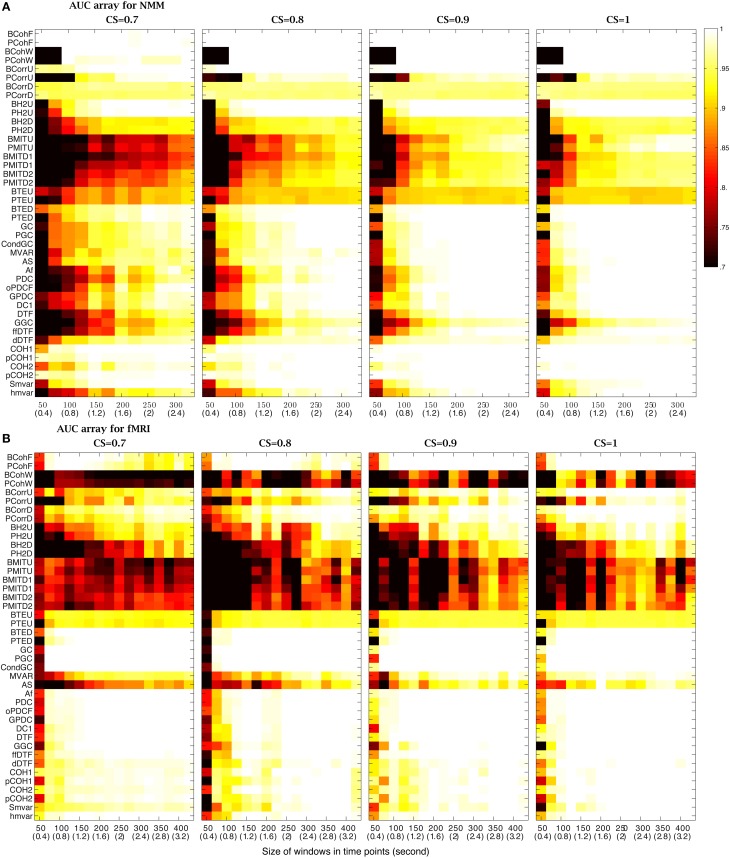
**Effect of the size of the analysis windows on the performance of the methods**. **(A)** AUC arrays of 42 methods for NMM signals as a function of window size [from 50 (0.4 s) to 800 (6 s)] for different connection strengths (CSs from 0.7 to 1). **(B)** AUC arrays of 42 methods for fMRI signals as a function of window size [from 50 (100 s) to 600 (1200 s)] for different connection strengths (CSs from 0.6 to 0.9). The color scale shows the corresponding AUC values.

Experimental datasets can be obtained with different sampling frequencies. In order to take this into consideration, the analysis platform uses time points rather than specific time units (these can be obtained by a simple conversion). We varied the size of sliding windows from 50 (0.4 s) to 800 (6.4 s) time points for NMM and from 50 (100 s) to 600 (1200 s) time points for fMRI datasets, testing different CS values. The sliding windows overlapped by half their length and the timeseries length was 8000 (64 s) time points for NMM and 5120 (10240 s) time points for fMRI. Figure 4A shows that for NMM signals with a strong connection strength, (CS= 1), 25 methods had an AUC= 1, when the size of widow was 600 (4.8 s) time points, while 7 methods were successful with a 150 (1.2 s) time point window. All methods failed with 50 (0.4 s) time points. Figure 4B shows that for fMRI signals with a strong connection strength (CS= 0.9), 28 methods had an AUC= 1 when the size of widows was set to 600 (1200 s) time points, while 6 methods were successful with a 50 (100 s) time point window.

Figure 4 also demonstrates that the size of the window needs to be increased to maintain performance as the CS decreased. For example, for fMRI signals, GC had an AUC= 1 for windows over 200 (400 s) time points when CS= 0.9. However, the window needed to contain at least 250 (500 s) time points when CS= 0.8 and 600 time points when CS= 0.6. In contrast, some methods were robust to variation of connection strength, such as, GC for NMM signals and BCohF for fMRI signals. It is interesting to note that BCohF performed best for small windows as CS decreases.

#### Number of windows

**Figure 5:**
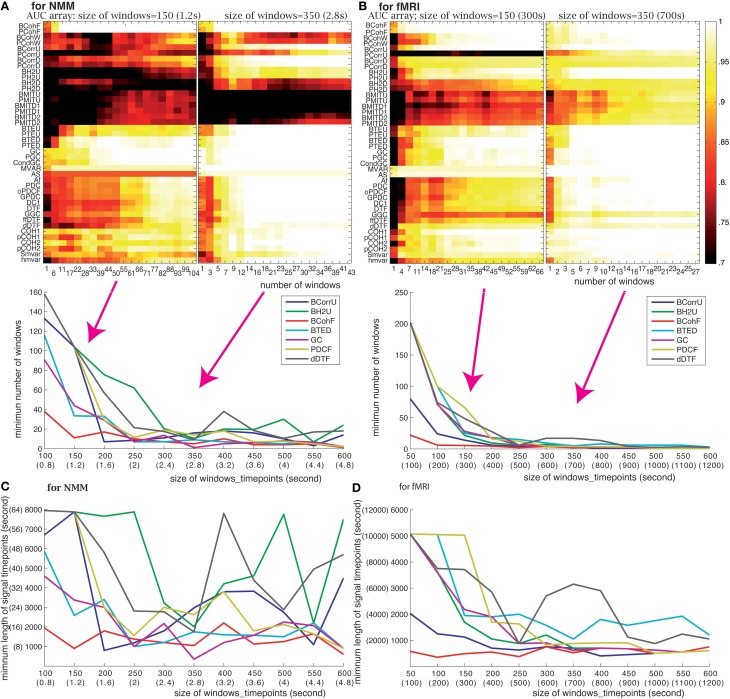
**Effect of the number of windows: (A) for NMM signals and (B) for fMRI signals**. The upper panels show the AUC arrays of 42 methods as a function of the number of windows for 150 (1.2 s for NMM and 300 s for fMRI) and 350 time point windows (2.8 s for NMM and 700 s for fMRI). The bottom panels show the minimum numbers of windows as a function of window size for 7 methods out of the 42. Panels **(C,D)** show the minimum length of analyzed signal (in time points) to find the correct structure as the function of window size for NMM and fMRI signals, respectively. The methods (color code) are the same as in the panel above.

We evaluated the effect of changing the number of windows using two window sizes: 150 (1.2 s for NMM and 300 s for fMRI) and 350 (2.8 s for NMM and 700 s for fMRI) time points. Figure 5 shows that the effect of window number on performance depends upon window size. As expected, the use of small size windows requires a larger number of windows to maintain performance. For example, GC needed a minimum of 50 windows for NMM and 38 windows for fMRI for a 150 time points windows, but 7 windows for NMM and 6 windows for fMRI when using 350 time point windows. We computed the minimum number of windows sufficient to identify the underlying structure as a function of the window size. The bottom panel of Figures 5A–B shows that the minimum number of windows (the corresponding AUC> 0.999) decreased as the size of the windows increased. For windows above 450 (3.6 s for NMM and 900 s for fMRI) time points, the minimum number of windows remained constant (between 10 and 20) for most methods.

#### Length of time series

The length of the time series is also a crucial factor. It needs to be as short as possible since, in real datasets, functional connectivity may change dynamically over time. The minimum length of signal can be directly derived from the previous analysis, and is shown in Figures 5C,D. For NMM signals, 2000 (16 s) time points were sufficient for GC, BTED, BCohF, PDCF, and dDTF methods when using a 350 (2.8 s) time point window. For fMRI signals, 1750 (3500 s) time points were sufficient for 5 chosen methods (BCohF, BCurrU, Bh2U, GC, and PDCF) for windows larger than 350 (700 s) time points.

#### Frequency bands

**Figure 6:**
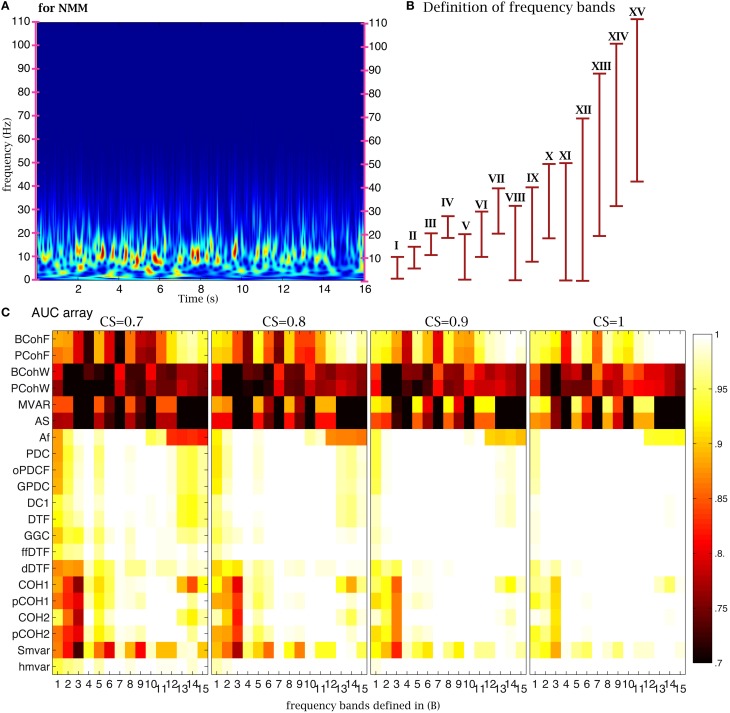
**Effect of frequency bands for NMM signals**. **(A)** Time frequency analysis of NMM signals using a wavelet transform. **(B)** Repartition of the 15 frequency bands. **(C)** Average AUC arrays of 21 methods (frequency domain) over 154 structures as the function of the frequency bands defined in **(B)** for different connection strengths.

**Figure 7:**
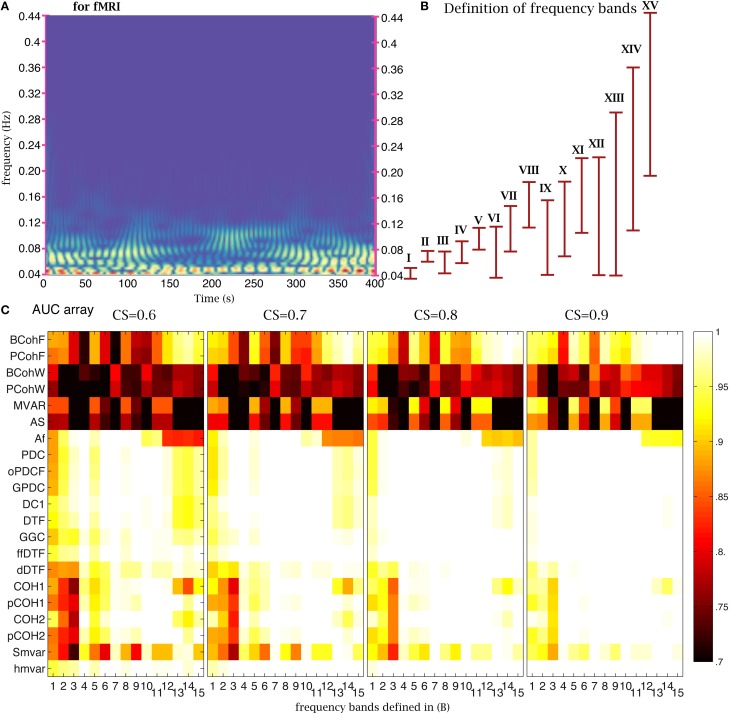
**Effect of frequency bands for fMRI signals**. **(A)** Time frequency analysis of fMRI signals using a wavelet transform. **(B)** Repartition of the 15 frequency bands. **(C)** Average AUC arrays of 21 frequency methods over 154 structures as a function of the frequency bands defined in **(B)** for different connection strengths.

One important parameter in coherence and 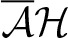 family methods is the frequency range. The time-frequency analysis of NMM signals revealed the presence of oscillations in the 2–20 Hz frequency band (Figure 6A). To test the sensitivity of the 21 coherence and 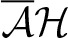 family methods to frequency bands, we defined 15 frequency bands (Figure 6B). Based on the previous analysis, we chose 4000 time points (32 s for NMM and 8000 s for fMRI) for the length of the time series, 350 (2.8 s for NMM and 700 s for fMRI) for the size of the window, and 21 windows. The corresponding AUC values are shown on (Figure 6C) for different values of CS. When the CS was very strong, the 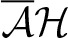 family was insensitive to frequency bands, in particular the 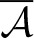 group. For example, although the signal was relatively poor in band XV, the true structure could be recovered. However, when CS decreased, performance decreased if bands in which the signal was expressed were missing. Figure 7 shows a similar analysis for fMRI signals, in which the main frequency was below 1 Hz (Figure 7A).

#### Maximum lags and model order

**Figure 8:**
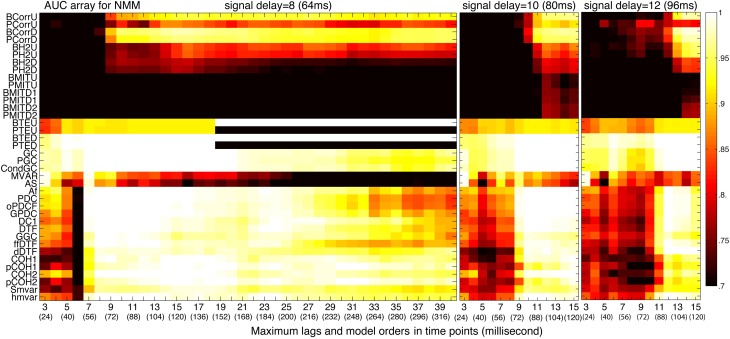
**Effect of the maximum lags and model orders**. From left to right, we considered datasets with different signal delays [from 8 (64 ms) to 12 (96 ms)]. Then, we calculated the average AUC arrays of 38 methods over 50 structures as a function of different computed delays (maximum lags for model-free and transfer entropy families and model orders for Granger and 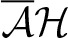 families).

The signaling between neurons and brain regions involves time delays, which must be taken into consideration in some analysis methods. Figure 8 shows the results when three different time delays were added to NMM signals. Other parameter values were 4000 time points (32 s) for the length of the time series, 350 (2.8 s) for the size of each of the 21 windows and frequency band VIII. The maximum lag is a key parameter for model-free methods in the time domain and the transfer entropy family (the coherence family does not have this parameter). Furthermore, the simulated time delay and lag parameter can interact in terms of determining performance. To illustrate this, methods with a maximum lag were used to evaluate the maximal connectivity by comparing connectivity values using lag values from 0 to the maximum lag. Figure 8 shows that transfer entropy was quite robust against the variation of both signal delays and maximum lag (provided that maximum lag ≥ signal delay).

Model order is a key parameter for Granger and 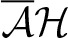 families in the presence of time delays. For the 3 different signal delays in Figure 8, the model orders used by Granger and 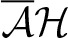 families need to be larger than or at least close to the signal delays. Note that, high model orders can cause these methods to fail. For example, for a signal delay of 8 (64 ms), the Granger and 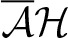 families were unable to infer the underlying structures for model orders >30 (240 ms). In addition, when the model order was very small (at 3 (24 ms) for three tested signal delays), some methods, such as GC, PGC, MVAR, were able to infer the underlying structures.

In summary, these evaluations—performed using the NMM and fMRI simulations—suggest that analysis parameters can have a profound effect on the ability of functional connectivity measures to recover the underlying effective connectivity. Most of the effects we described above can be understood intuitively. However, the main contribution of these analyses is to note that, using simulated data, the parameters can be optimized for any empirical data set at hand. MATLAB code is available to reproduce the analyses reported above—to find the valid parameter range for any functional connectivity measure or configuration of the generative models. Using appropriate parameters, we now turn to the relative performance of the various measures on different simulated models.

### Relative performance

Using the parameter values discussed in the previous section (Supplementary Table 2), we systematically investigated the performance of each of the 42 methods on the 154 possible structures, for different CS values. Intuitively, weak connection strengths may be difficult to detect. In reality, CS may take any value over different connections. Here, we used the same CS values for all links so that we could investigate the sensitivity of methods on the different CS levels.

**Figure 9:**
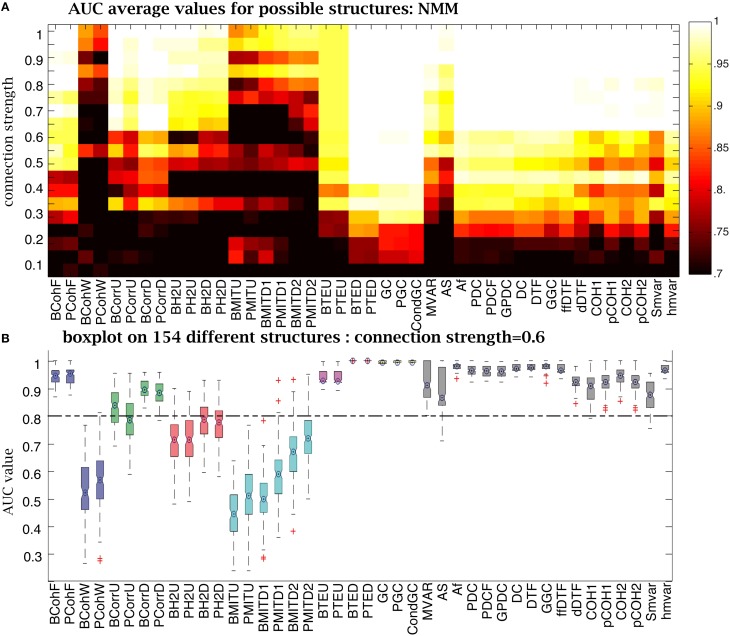
**Performance of the 42 methods for NMM signals for different connection strengths**. **(A)** Average AUC arrays of 42 methods over different structures as a function of connection strength. **(B)** Distribution of AUC values for all 154 structures given by the 42 methods when the connection strength is set to 0.6. The central marks are the median, the edges of the box are the 25 and 75th percentiles, the whiskers extend to the most extreme data points not considered outliers, and outliers are plotted individually (plus signs).

For the NMM simulations, when CS was strong, (above 0.9) most methods had an AUC= 1 (Figure 9A). But as CS decreased, the number of failures rose. Ranking the different methods as a function of their sensitivity to CS (from least to most) gives: Granger family, transfer entropy, 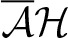, correlation, coherence, h^2^. Figure 9B uses boxes to show AUC values distribution over 154 structures for the 42 methods for a CS of 0.6. Granger and transfer entropy were able to recover the underlying structures. When comparing the other methods with an AUC> 0.8, the correlation and coherence families were more sensitive to variation of the structures than the 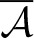 group from 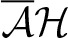 family.

**Figure 10:**
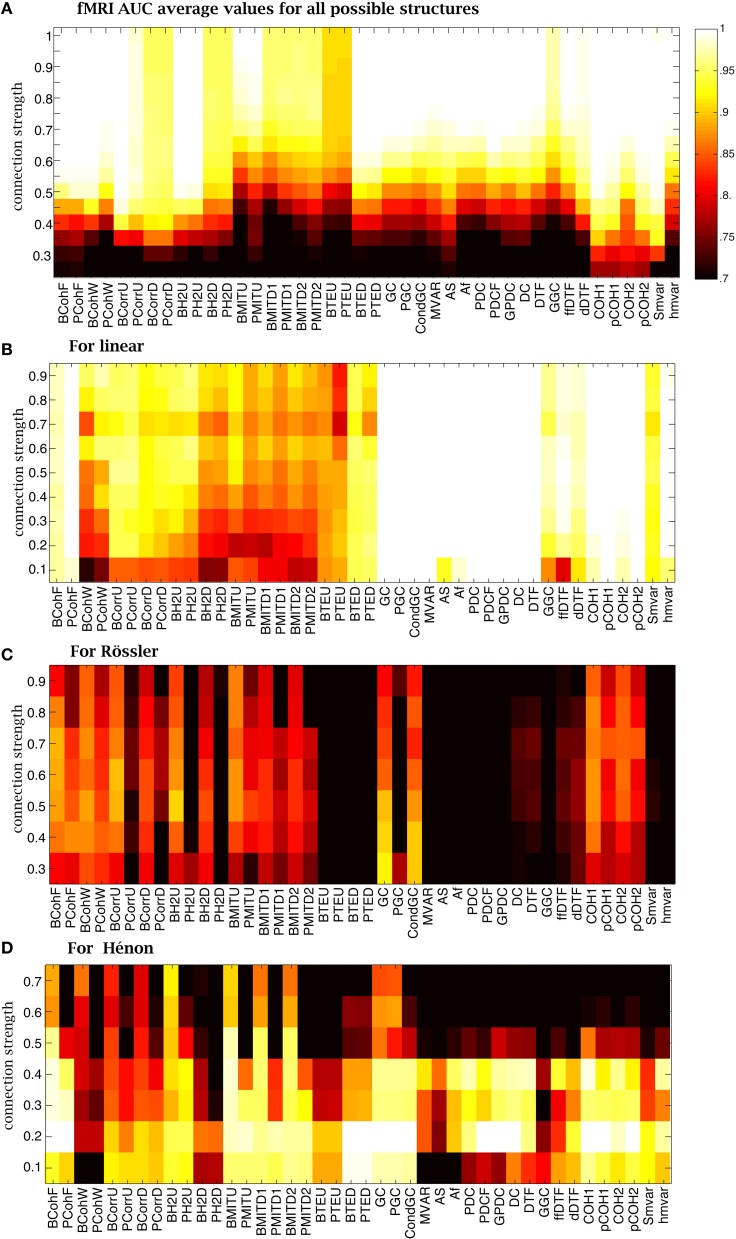
**Average AUC arrays of the 42 methods over different structures as a function of connection strength for (A) fMRI, (B) linear (C) Rössler and (D) Hénon**.

For fMRI simulations, when the CS was strong, more methods had an AUC= 1 relative to the NMM simulations, as shown in Figure 10A. The wavelet coherence and the mutual information family performed well on fMRI, while they failed on NMM signals. In Supplementary Figure 3A, using a CS of 0.8, we could find methods in each family, which were able to recover connectivity structures. For all other methods with median AUC< 1, the methods were robust against the variation of structures because the range of AUC metrics was small.

The NMM and fMRI simulations are biologically plausible. In order to get a broader overview of the performance of the methods, we used abstract (mathematical) generative models to determine their sensitivity to the nature of the underlying dynamics and connectivity (linear vs. non-linear). The analysis of linear systems revealed that nearly all methods were robust for different CS values (Figure 10B). The Granger and 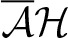 families were able to recover ground-truth connectivity structures in most cases. In contrast, Correlation, h^2^ and mutual information families mostly failed. Supplementary Figure 3B shows that coherence, Granger and 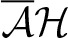 families were robust against the variation of connectivity structures, while all other methods were quite sensitive to the variation of structures at a CS of 0.4.

When considering Rössler systems, which are strongly non-linear systems with linear coupling, we found that all families performed poorly (Figure 10C). The performance of the methods was not linearly dependent on the CS. For example, the Granger family performs better for a CS of 0.3 than for a CS of 0.9. The coherence family performed better for CS between 0.5 and 0.7. The box-plot in Supplementary Figure 3C shows that the AUC values were very sensitive to the variation of the connectivity structure for the Rössler dynamics, because the distribution of AUC values was widespread. Some model-free methods, such as PCohW, BCorrU, Bh^2^U, and BMITU, were able to infer the underlying undirected structures for some structures, but failed on others. Transfer entropy and most methods from 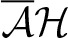 family failed for the Rössler simulations.

Finally, in the case of Hénon systems, which are non-linear systems with non-linear coupling, we found that most methods failed for CS values larger than 0.5 (Figure 10D). The best performance was obtained for CSs around 0.2. For example, coherence, transfer entropy, Granger and 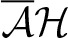 family were able to recover all underlying connectivity structures. Mutual information and h^2^ families performed better than correlation family. Supplementary Figure 3D shows that all methods were more robust against variation of structures relative to Rössler systems.

### Effect of noise

Recorded signals include both system noise and observation noise. System or state noise refers to endogenous or random fluctuations of unobserved (hidden) states. Observation noise refers to the noise caused by the equipment, the environment etc. In other words, system noise generates the signal through the generative model, while observation noise is generally added to the signal to produce observed data.

**Figure 11:**
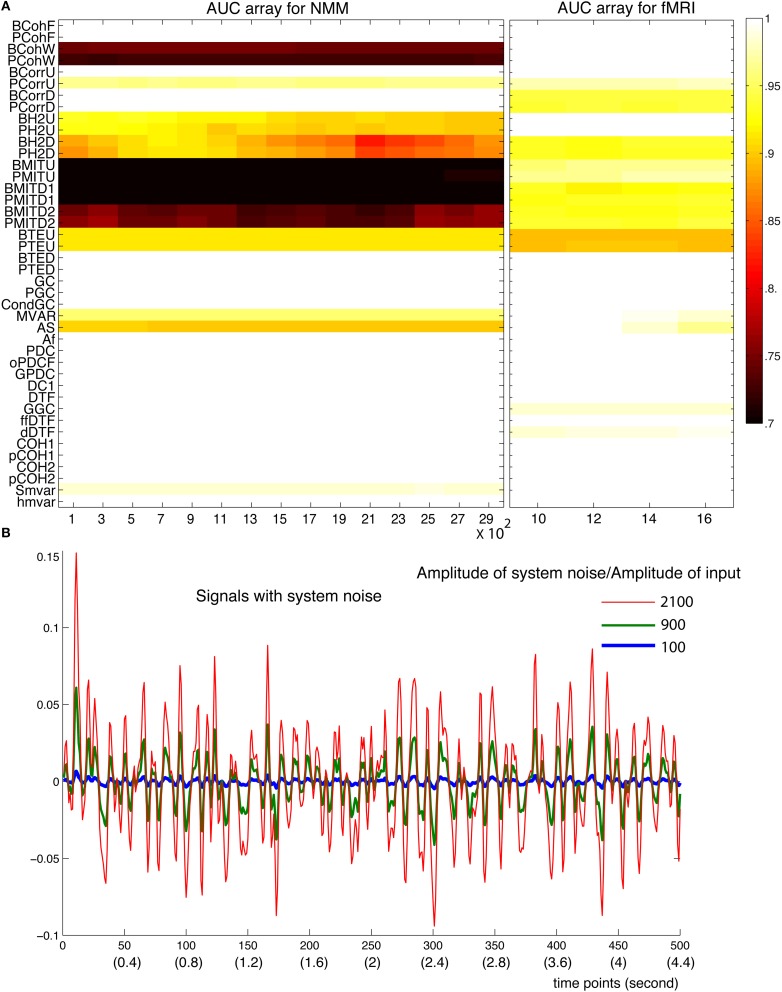
**Robustness of the methods against system noise**. **(A)** AUC array of the 42 methods as a function of the ratio between the amplitude of system noise and the amplitude of inputs for both NMM (left) and fMRI signals (right). **(B)** Examples of signals generated with different amplitudes of system noises (100, 900, and 2100 times more than the amplitude of the input signals).

In the NMM, spiny stellate cells are driven by exogenous inputs containing both white (with a flat frequency spectrum) and pink noise (frequency density proportional to f^−1^). To mimic system noise, we added white and pink noise to the hidden states of the NMM, which we called NMM system-like noise (green curves in Supplementary Figure 4). For fMRI, system noise contained both white and brown spectral components (red curves in Supplementary Figure 4), brown noise has a density proportional to f^−2^. The level of system noise was quantified as the ratio between the amplitude of system noise over the amplitude of deterministic input. Figure 11 shows that most methods were stable up to a ratio of 2900 for NMM and 16 for fMRI. Out of these ranges, the simulation models became unstable. This reflects the fact that system noise contributes to the signal and discloses functional dependencies or connectivity among neuronal sources. In fact, such system-like noise can be regarded as exogenous inputs. In another word, the strength of exogenous input determined the amplitude of the signals but did not change the coupling of signals.

In real datasets, experimental procedures may cause observation noise and also the environment can constitute a major difficulty for data analysis. After generating NMM or fMRI signals, we added four types of observation noise to each recording site (node). We considered white and pink noise—and the specific case where the noise has the same spectrum as the model input, i.e., mixtures of white and pink noise and mixtures of white and brown noise. Finally, we considered two cases: when observation noise was the same for all nodes (e.g., 50–60 Hz for EEG), or when it varied over nodes (e.g., each sensor at each node had specific signal-to-noise properties). We used the signal-to-noise ratio (SNR= 20log(Amplitude-of-single/Amplitude-of noise)) to quantify the level of noise. When each channel had a different noise level, we added the noise to each channel with the same SNR in order to investigate the robustness of methods against different noise levels. For completeness, we also considered the case in which each channel receives a different noise level with different SNRs (Supplementary Figure 5). The mathematical details are in Supplementary Section 1 and Supplementary Figures 1, 2, 4.

**Figure 12:**
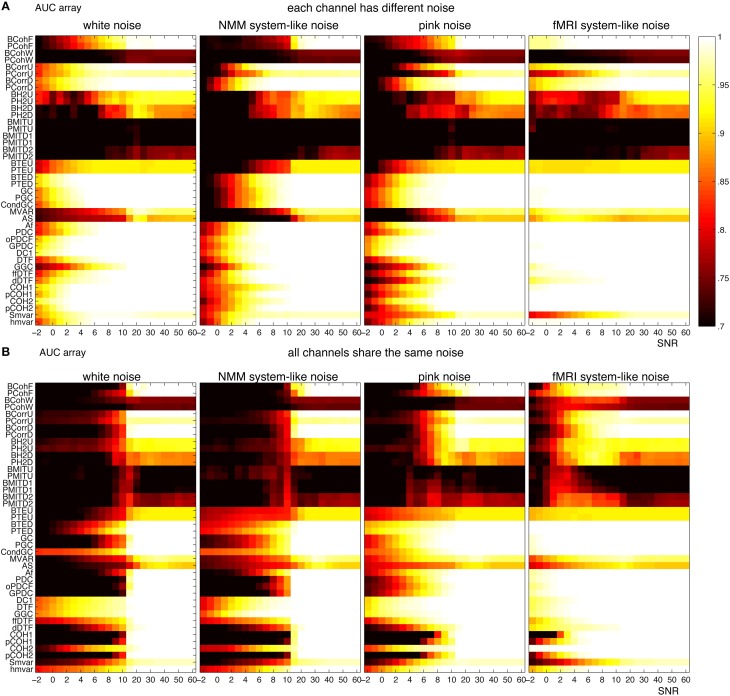
**Robustness of the 42 methods against the observation noise for NMM signals**. The figure shows average AUC arrays of the 42 methods as a function of the SNR (signal-to-noise ratio). **(A)** Each channel receives a different noise **(B)** All channels receive the same noise.

For NMM, Figure 12A shows that the methods were more robust to white and brown mixtures than white and pink mixtures, when each channel had a different noise. The possible reason is that the spectrum of pink mixtures was more similar to the spectrum of the input. When all channels shared the same noise Figure 12B, the methods were more robust against pink and brown noise than to white and pink noise. Interestingly, in general, the methods were more accurate when each channel had a different noise.

**Figure 13:**
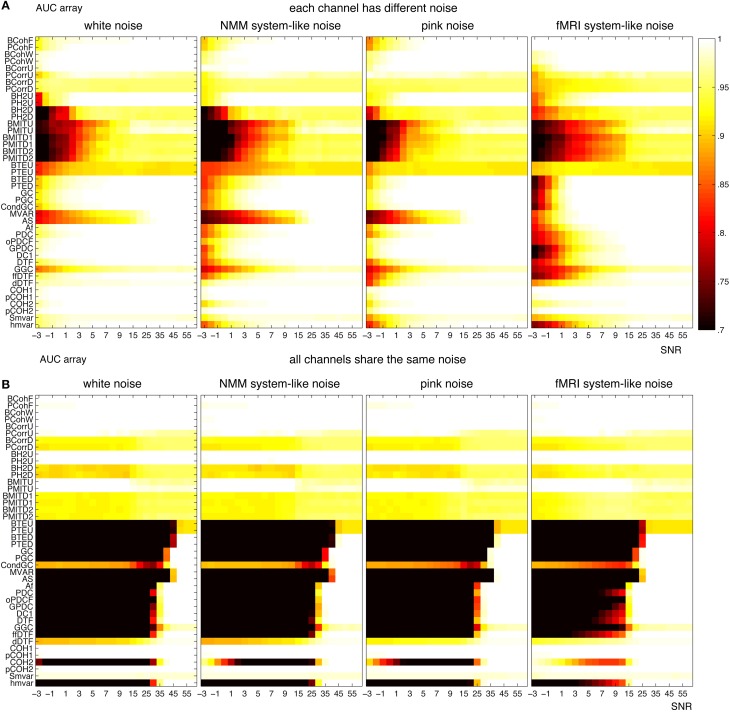
**Robustness of the 42 methods against the observation noise for fMRI signals**. The figure shows average AUC arrays of the 42 methods as a function of SNR. **(A)** Each channel receives a different noise **(B)** All channels receive the same noise.

For fMRI, Figure 13A shows that several methods (from coherence, correlation and 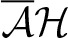 families) could find the underlying structures even for SNR= −3 (i.e., the amplitude of noise is 1.4 times of the amplitude of signals), when each channel had a different noise. The robustness of the methods can be ordered from high to low: white, pink, pink mixtures and brown mixtures. Figure 13B shows similar results when all channels shared the same noise. However, all direct methods failed when SNR< 15. Figure 13B shows that transfer entropy, Granger and 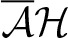 families were more accurate when each channel had a different noise, whereas the coherence, correlation, h^2^ and mutual information families were more accurate when channels shared the same noise.

When each channel had a different noise level with different SNR, we found that performance was a function of maximal SNR (Supplementary Figure 5). Because the noise levels of most channels is less than the maximal SNR, most methods (from coherence, correlation, h^2^, transfer entropy, Granger families) shows better performance for a given SNR when channels had a different SNR noise (except brown mixtures) relative to a different noise with same SNR. The directed methods from 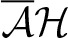 families were more sensitive when each channel has different noise and different SNR.

**Figure 14:**
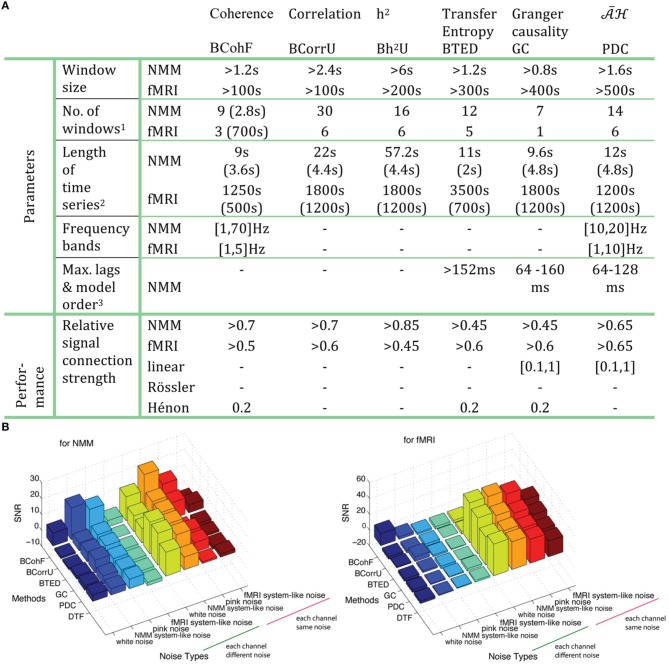
**(A)** The optimized parameters and relative performance are summarized for 6 chosen methods, each from 6 different families. Note that 1: Numbers of windows are obtained by taking a 2.8 s window for NMM and 700 s for fMRI. 2. The first line is the length of time series while the second line shows the window size. Both numbers of windows and length of time series are based on the AUC> 0.999. 3. Results are obtained for a signal delay of 8 (64 ms). **(B)** The minimal SNR for 8 types of noise by 6 chosen methods on both NMM and fMRI according to AUC> 0.95.

Figure 14 summarizes the appropriate parameter ranges for 6 specific methods, relative to signal connection strengths and the minimal SNR, which can successfully retrieve the ground truth graphs. Figure 14B also clearly demonstrates that model-based methods are more robust when each channel has a different level of noise vs. the case where the noise is shared by all channels.

### Computational cost

**Figure 15:**
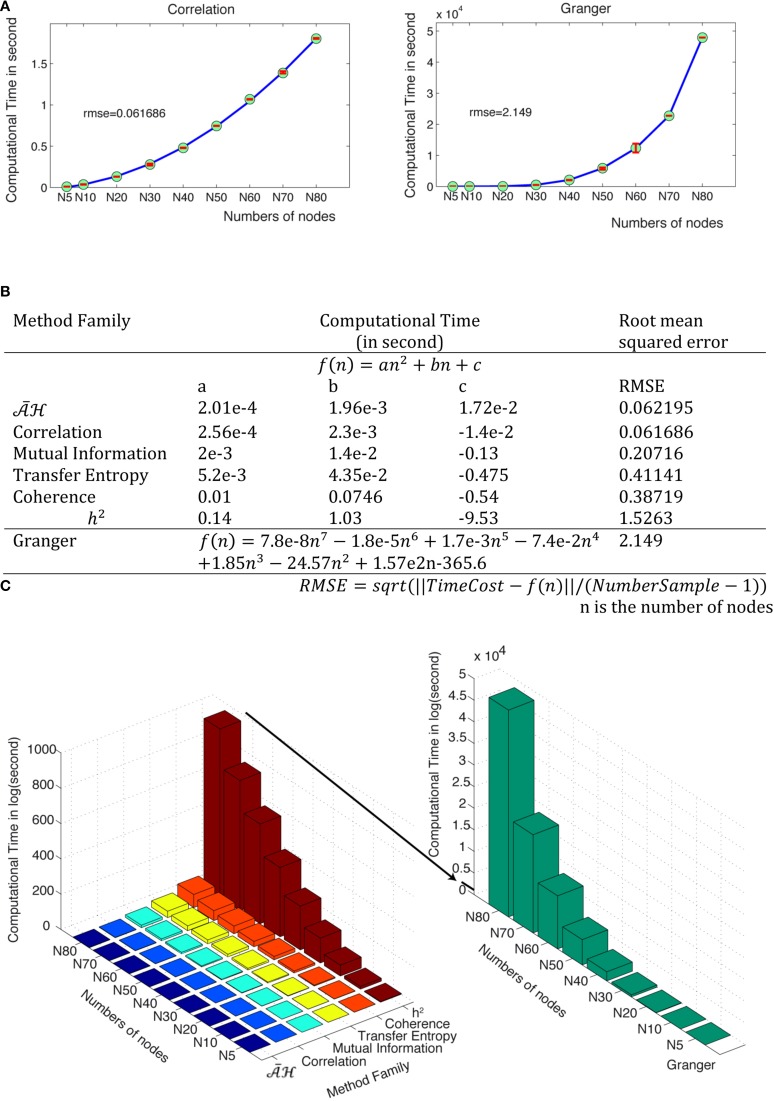
**Computational time for the different method families**. **(A)** The computational time (in seconds) is a function of the number of nodes for correlation and Granger families. Green points are the tested values and red bars show the distributions from the different trials. The fitted curves are shown in blue and the mathematical equations for all method families are shown in **(B)**. **(C)** Computation time in second as a function of numbers of nodes and method families.

Computational complexity can be a limiting factor when performing network analysis, especially when the size of the network increases. We measured the computational time of each method family for an analysis performed in a 350 time points window on an Intel Xeon E5–2690 when the number of nodes increases from 5 to 80. Figure 15A shows the computational time in seconds for correlation and Granger families. Other families are available in Supplementary Figure 6A. The complexity cost for Granger is O(n^7^), where n is the number of nodes, whilst the computational cost for all other method families is O(n^2^) but with different parameters. Figure 15B provides the estimation equations of computational time for a given number of nodes. The computational time cost of the discussed methods families can be ordered from low to high: 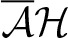, correlation, mutual information, transfer entropy, coherence, h^2^ and Granger (Figure 15C and Supplementary Figure 6B).

## Discussion

We studied these 42 methods because they cover a wide range of approaches for characterizing functional connectivity. Of the 42 methods, 28 assume underlying linear models of statistical dependencies; 4 (h^2^ family) use piece-wise linearization and the last 10 methods (mutual information and transfer entropy) use entropy measures to account for nonlinearities. Within the Granger and 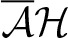 families, various methods use different statistical estimation methods, although they are all based on linear multivariate autoregressive models.

Since functional connectivity analysis is routinely performed on electrophysiological, encephalographic and fMRI signals, we selected neural mass models and associated fMRI models to generate datasets. However, when they were first introduced, most functional connectivity methods were tested on (biological) mathematical models. For example, the Granger family (Seth, 2010) and 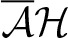 (Baccalá and Sameshima, 2001) families were tested on linear models, while non-linear methods were tested on non-linear models (Lopes da Silva et al., 1989). In light of this, we considered both biologically plausible generative models and more abstract mathematical models with linear and non-linear dynamics. Any other method and/or other generative model can easily be introduced in the evaluation framework.

### Parametric optimization

Both evaluation and application of a functional connectivity method require valid parameters. The proposed framework attempts to provide a systematic way to find the valid parameters. After choosing candidate methods, it is necessary to select a model, which is related to the simulated data. Using the provided code and referring to parameter ranges presented here, it is then possible to choose the possible parameter ranges for the candidate methods on the simulated data. One can thus find the valid parameters. Note that we assumed that the performance of connectivity analysis methods has the same characteristics on both empirical data and the simulated data.

Recovering the underlying (effective) connectivity graph requires sufficiently long time series. Our results suggested using a window size larger than 350 time points and at least 15 windows (with a half-window overlap) are necessary for most methods. Thus, for most methods, 4000 (32 s) time points should be sufficient for NMM and 3000 (6000 s) time points for fMRI time series. Such long acquisition times are difficult to implement for fMRI; e.g., when analyzing resting state networks. However, some methods, like bivariate coherence Fourier (BCohF) perform well with short duration acquisitions (Figure 5D). Furthermore, it should be noted that connectivity averages—based on multiple short runs or subjects—often converge to meaningful values (as we demonstrate in the sliding window approach in the current paper and using simulations in Friston et al., 2014). It is important to note that using a single long time window can be unreliable. Both the number of windows and their length are critical parameters. These considerations are valid if the connectivity strength does not change during this period. However, brain activity is highly dynamical, with functional links between regions/neurons being permanently turned on and off in a brain state-dependent manner. Indeed, in the dynamic causal modeling of effective connectivity, it is this context-sensitive (bilinear) change in connectivity that is usually of primary interest (Friston et al., 2003; Fox et al., 2005). When the changes in effective connectivity are not under experimental control, this can represent a challenging problem, which we hope to consider in future work. In the present context, the current analyses suggest that, if changes in functional connectivity have time constants greater than about 4000 time points, it should be possible to identify the connectivity graph and its fluctuations over slower time scales.

The appropriate maximum lag and model orders for autoregressive and embedding methods must be at least in the range of signal delays. However, large maximum lags or model orders do not necessarily provide more accurate results. Estimating signal delays in real datasets is a difficult task—although it is done routinely in dynamic causal modeling of EEG data. In the analysis of functional connectivity, one possibility would be to try all maximum lags and model orders and determine whether different methods give consistent results.

### Performance of the methods

Although our goal was not to understand why methods succeed or fail to recover graphs, our systematic analysis of 42 methods provide some clues regarding their performance. In the case of model-based methods, the Granger family recovered most underlying structures. Barnett et al. (2009) show that, in theory, Granger causality and transfer entropy are equivalent for Gaussian variables. In the present study, we demonstrated that transfer entropy was as good as Granger family in most cases, except for linear and Rössler systems. The 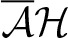 family also performed quite well on most datasets (except Rössler systems), but was not robust to variations in connection strength.

In the case of model-free methods, Fourier coherence performed quite well for recovering undirected graphs. Wavelet coherence only performed well on fMRI datasets. The correlation family performed slightly better than h^2^ on NMM and fMRI, but worse on non-linear mathematical models (Rössler and Hénon). Mutual information methods only worked on fMRI datasets when the connection strength was strong (and on Hénon systems).

The relative performance of the methods we considered also depends upon the structure and complexity of the signal. It is generally assumed that biological systems are inherently non-linear. All methods performed better on the neuronal models (NMM and fMRI) than on the purely mathematical non-linear models (Rössler and Hénon). Most methods also performed better on fMRI than on NMM. One possible reason is that the number of hidden states within each node of a NMM (13 states) is larger than for fMRI (1 state). Another possible reason is that NMM has a higher degree of nonlinearity than fMRI. The degree of nonlinearity can be defined as the degree to which a linear surrogate can approximate a non-linear system, at least for a finite time. Two reasons may explain why the methods performed better on Hénon than on Rössler: The Hénon system has 1 hidden state while the Rössler has 3 states; furthermore, the Rössler system has a larger degree of nonlinearity than the Hénon system.

A recent study investigated different methods using simulated fMRI data (Smith et al., 2011). We used the most stringent criterion: AUC= 1, which would correspond to c-sensitivity= 1 in Smith et al. (2011). In the latter study, even the best performing methods did not reach this level, whilst in our case, many methods succeeded with an appropriate choice of parameters. In order to assess the possible origin of these differences, we analyzed the datasets used in Smith et al. (2011) (Supplementary Figure 7 and parameters in Supplementary Table 2). Differences with the present study can be noted: for example, undirected methods from both correlation and coherence families can identify the exact truth structures, while these methods failed in Smith et al. (2011). This discrepancy may stem from the fact that we performed a systematic parametric search to identify the optimal parameter range for each method. This protects against false negative conclusions (i.e., when a method fails due to an inappropriate parameter choice). Second we averaged the results across the sliding windows rather than using a single window. Directed methods such as Granger and PDC methods performed poorly for the datasets in Smith et al. (2011) perhaps because the connectivity strengths maybe too low to be detected by GC in Smith et al. (2011). This is consistent with our analysis (Figure 10A): GC failed when the connectivity strength was too low.

### Influence of fluctuations and noise

System noise, which has a role that is similar to that of exogenous input, did not affect the performance of the methods. In contrast, all methods were sensitive to observation noise, in particular when its spectrum was similar to the exogenous input. Model-based methods were more robust when each channel had different noise, while model-free methods are more robust when all channels shared the same noise. We note that many signal-processing techniques have been designed to eliminate a stable source of noise common to all channels and may be usefully applied in this context.

### Influence of connection strength

Intuitively, if two nodes are strongly connected, finding an edge between these two nodes is a relatively easy task. However, when the connection strength became very weak, all methods failed. For intermediate values, the ability to identify the link was method-dependent. For example, the Granger and transfer entropy families were better at detecting weak links for NMM, but the correlations, coherence, and h^2^ families performed slightly better for fMRI.

### Varying number of edges, hidden nodes, and non-stationarity

Our analysis is based on networks comprising 5 nodes and 5 links. However, our (open source) MATLAB code allows one to vary the number of nodes and links, as well as the architecture of the network. The appropriate range of parameters (in particular the size of the sliding window) can then be optimized for any particular architecture defined in terms of graph size.

When analyzing an experimental dataset, part of the real network could be hidden from observation. The framework presented in this paper allows one to characterize the influence of hidden nodes on connectivity estimates. For example, in fMRI, it is possible to simulate data with and without the influence of (hidden) nodes and compare the associated connectivity estimates.

Non-stationarity is common in experimental datasets. The good performance of some methods presented in the present study may stem from the stationary nature of the simulated data we have used. Some of these methods may perform poorly in non-stationary situations, which would require explicit modeling. In principle, the present framework allows one to test the robustness of methods to violations of stationarity assumptions—by comparing the estimates based on stationary and non-stationary simulations. We hope to address this particular issue in a future paper.

## Conclusion

The pragmatic contributions of this work are to illustrate how one can optimize analysis parameters and to explore the dependency of various functional connectivity measures on the underlying generative process and architecture. The particular analyses presented in this paper illustrate the optimization under some particular generative models. We hope that people will use the MATLAB code that we have made available to reproduce this sort of analysis with a focus on specific issues of interest; for example, the impact of variable haemodynamic delays over regions or nodes. This can be implemented simply by supplying node specific haemodynamic parameters to the fMRI simulations. Clearly, there are many other variations of the generative models and functional connectivity measures one might consider, using the procedures described above.

Our results demonstrate that no single method is optimal for all types of data. We propose that combining methods, knowing when they fail and when they succeed, is the obvious strategy. In our next communication using the framework established in this paper, we will present a MULtiple connectivity ANalysis (MULAN) algorithm. This scheme enables one to identify the most probable connectivity structures by integrating the results of the current methods. Subsequently, we will describe an extension that allows connectivity graphs to evolve dynamically.

The schemes described in this paper are implemented in Matlab code and are available freely from in github: https://github.com/HuifangWang/MULAN.

### Conflict of interest statement

The authors declare that the research was conducted in the absence of any commercial or financial relationships that could be construed as a potential conflict of interest.

## Footnotes

^1^ http://ins.medecine.univmed.fr/cross-cutting-research/the-virtual-brain-project/tvbhpc/.
