# Supplementary material for: A systematic framework for functional connectivity measures
Source: Front Neurosci. 2014 Dec 9;8:405. doi: 10.3389/fnins.2014.00405 (PMC4260483; doi:10.3389/fnins.2014.00405)
Supplement: Supplementary file 1 [file Presentation1.PDF]

Frontiers in NEUROSCIENCE

Method Article
8 November 2014

Supplementary Material: A systematic framework for functional connectivity measures

Huifang E Wang [1,2,*], Christian G Bénar [1,2], Pascale P Quilichini [1,2], Karl J Friston [3], Viktor Jirsa [1,2] and Christophe Bernard [1,2,*]

[1] *Aix Marseille Université , INS, Marseille, France*
[2] *Inserm, UMR-S 1106, Marseille, France*
[3] *The Wellcome Trust Centre for Neuroimaging, University College London, Queen Square, London, UK*

Correspondence*:
Christophe Bernard and Huifang E WANG
Institut de Neurosciences des Systèmes, INSERM U1106, Aix-Marseille Universit, Marseille, 13005, France., christophe.bernard@univ-amu.fr and elizabethhw@gmail.com

1 MATHEMATICAL DEFINITIONS OF SIMULATED SYSTEMS

Let $x_i(t)$ be the signal at node i at time t, with $i = 1, \cdots, n$, where n is the number of nodes and $\xi_i(t)$ is the noise at node i at time t. Let τ be the time delay. C_{ij} is the connection strength from j to i.

1.1 NMM MODELS

The details of the mathematical definition of the NMM model can be found in **Supplementary Figure 1**, (**David et al.**, 2006; **Moran et al.**, 2013). Thirteen neural states include currents $i_0 - i_5$ and membrane potentials $v_1 - v_7$ of three cell subpopulations: spiny stellate cells, pyramidal cells and inhibitory interneurons. For simplicity, time t and node i are omitted. System noise ξ^s is added to the current of the spiny stellate cells and observation noise ξ^o is added to the signal x_i. The sigmoid function is defined as $S(x) = 1/(1 + e^{2-x}) - 1/(1 + e^2)$. The main parameters are: $K_e = -4\,ms^{-1}$, $K_i = -16\,ms^{-1}$, $H_e = 8mV$, $H_j = 32mV$, $\gamma_{1,2,3,4,5} = \{128, 128, 64, 64, 4\}$. The input U comprises signals containing both white and pink noise, with amplitude 0.0865. The original codes are from `http://www.fil.ion.ucl.ac.uk/spm`. ξ^s has amplitude $[0.865, 250]$, and ξ^o has SNR from $[-3, 60]$. When each channel had a different noise, ξ^o is different from node to node. We used $C_{ij} \in [0, 1]$. We used 125 Hz to sample the NMM data and we also added the signal delay from 64 ms to 96 ms.

1.2 FMRI MODELS

The details of the mathematical definition of the fMRI model can be found in **Supplementary Figure 2** (**Friston et al.**, 2003). For each region, neural activity z causes an increase in vasodilatory signals s. Inflow f responds in proportion to s with concomitant changes in blood volume v and deoxyhemoglobin content q. A is the matrix with diagonal elements set to -0.5, and B is the matrix with diagonal elements of 0.0625. Within the hemodynamic state equations, $E(f, \rho) = 1 - (1 - \rho)^{1/f}$, $k = 0.64$, $\gamma = 0.41$, $\beta = 0.98$, $\alpha = 0.32$, $\rho = 0.34$. The BOLD signal is a function of v and q, with $V_0 = 0.02$, $k_1 = 7\rho$, $k_2 = 2$, $k_3 = 2\rho - 0.2$. The input U comprises signals containing both white and brown noise, with amplitude

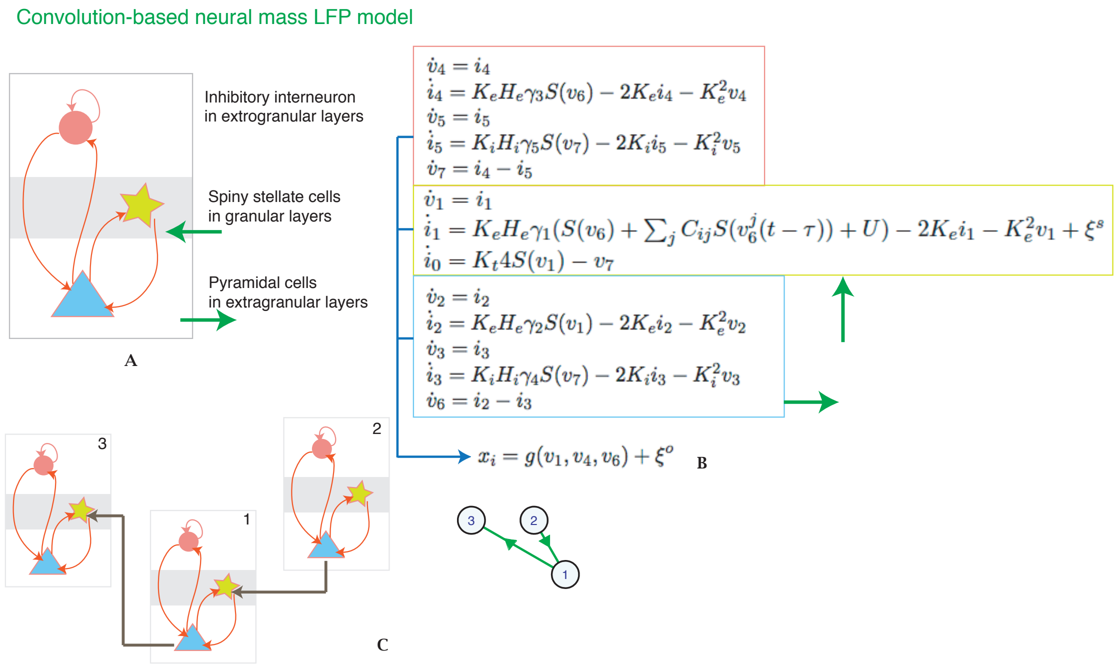

Supplementary Figure 1. Definition of convolution-based neural mass LFP models, adapted from (**Moran et al.**, 2013) (A) Three cell subpopulations include spiny stellate cells in granular layer IV, pyramidal cells and inhibitory interneurons in extra granular layers (II and III; V and VI). (B) Neuronal states include currents, i, and membrane potentials v. Extrinsic connections enter at granular cortical layers from pyramidal cells in extra granular layers of other subpopulations. (C) Example of the connectivity between the three subpopulations.

3.3482. The orignal codes are from `http://www.fil.ion.ucl.ac.uk/spm`. The BOLD data was sampled with a repetition time of $2s$. ξ^s has amplitude $[33.482, 5.3571]$, and ξ^o has SNR from $[-3, 60]$. We used $C_{ij} \in [0, 1.6]$, which is normalized to $[0, 1]$ for CS.

1.3 LINEAR SYSTEMS

For linear systems, the simulated data is produced by the following linear equations:

$$\dot{x}_i(t) = \sum_\tau \sum_j C_{ij}(\tau) x_j(t-\tau) + \xi_i(t),$$

with $C_{ij}(\tau)$ the coupling strength from j to i with time delay τ. In this study, we used $C_{ij} \in [0, 1]$.

1.4 RÖSSLER SYSTEMS

For Rössler systems, each node is based on a Rössler attractor (**Rössler**, 1979) and linearly coupled with equations:

$$\begin{array}{l} \dot{x}_i(t) = -a y_i(t) - z_i(t) + \xi_i(t) + \sum_\tau \sum_j C_{ij}(x_j(t-\tau) - x_i(t)) \\ \dot{y}_i(t) = -a x_i(t) + d y_i(t) \\ \dot{z}_i(t) = b + z_i(t)(x_i(t) - c)) \end{array} .$$

The system state for node i is $[x_i, y_i, z_i]$. In this study, we used $a = 0.15, b = 0.2, c = 10$, $d \in [0.98, 1.02]$ and $C_{ij} \in [0, 1]$.

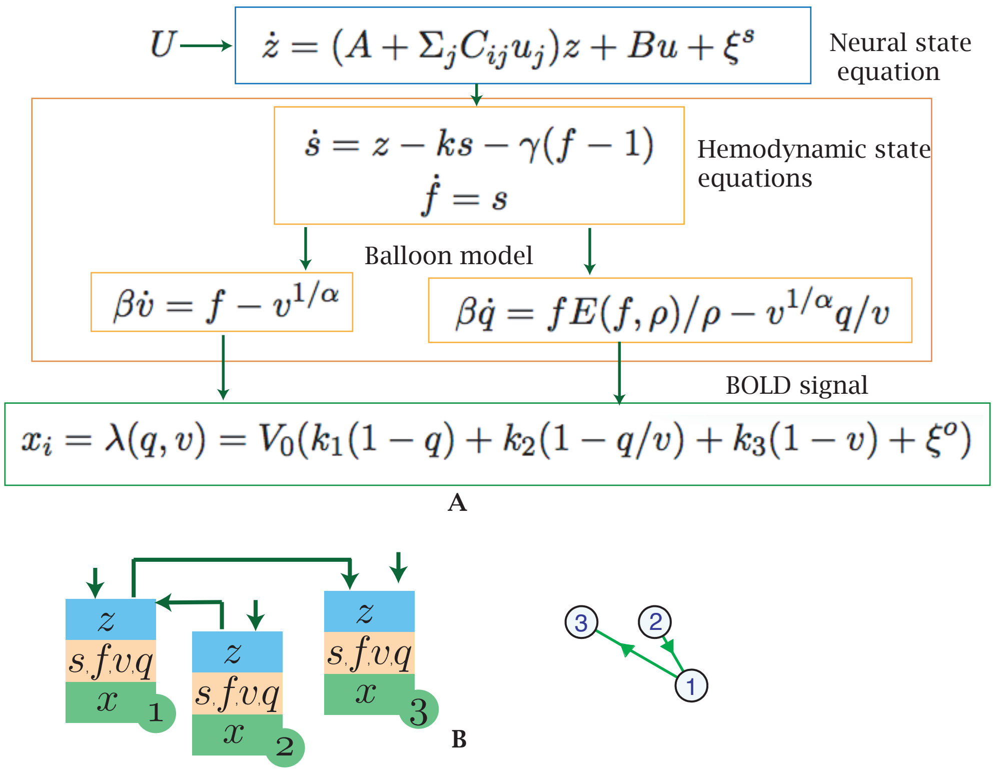

Supplementary Figure 2. Definition of DCM for fMRI models. (A) The bilinear neural activity x induces a vasodilatory signal s that increases blood flow f. Blood flow causes changes in volume v and deoxyhemoglobin q. These two hemodynamic states result in a predicted BOLD response y. Adapted from (**Friston et al.**, 2003) (B) Example for the connectivity between three channels of BOLD signals.

1.5 HÉNON SYSTEMS

For Hénon systems, each node was generated by Hénon mappings (**Hénon**, 1976) and nonlinearly coupled with the following equations:

$$x_i(t+1) = 1.4 - \sum_j C_{ij} x_i(t) x_j(t) - (1 - \sum_j C_{ij}) x_i^2(t) + b x_i(t-1) + \xi_i(t+1)$$

with $b \in [0.2, 0.3]$ and $C_{ij} \in [0, 3]$, which is normalized to $[0, 1]$ for CS.

2 MATHEMATICAL DEFINITIONS OF CONNECTIVITY ANALYSIS METHODS

Let $cov(\cdot, \cdot)$ be the covariance of the two variables and $var(\cdot)$ be the standard deviation of the variables.

Supplementary Table 1. Mathematical definitions of connectivity analysis methods

**Methods**	**Mathematical definition**
**Correlation**	Pearson correlation coefficient with delay (**Jovanović et al.**, 2013)
`BCorrD`	Bivariate correlation directed: $r_{i,j} = max_\tau cov(x_i(t), x_j(t+\tau)/(var(x_i(t))var(x_j(t+\tau))))$

PCorrD	Partial correlation directed: $r^P_{i,j} = -P_{i,j}/\sqrt{P_{i,i}P_{j,j}}$ with $P = [r_{i,j}]^{-1} = [P_{i,j}]$
BCorrU	Bivariate correlation undirected: $\bar{r}_{i,j} = max\{\lvert r_{i,j}\rvert, \lvert r_{j,i}\rvert\}$
PCorrU	Partial correlation undirected: $\bar{r}^P_{i,j} = -P_{i,j}/\sqrt{P_{i,i}P_{j,j}}$ with $P = [\bar{r}_{i,j}]^{-1} = [P_{i,j}]$
**h²**	The h^2 family starts from a scatter plot of x_i and x_j. Divide x_i into L bins; for each bin l, the average values $\bar{x}_{i,l}$ and $\bar{x}_{j,l}$ of x_i and x_j are calculated. Then the curve of regression is approximated by a line connecting the points $(\bar{x}_{i,l}, \bar{x}_{j,l})$ and $(\bar{x}_{i,l+1}, \bar{x}_{j,l+1})$: $\breve{x}_j = (\bar{x}_{j,l+1} - \bar{x}_{j,l})(x_i - \bar{x}_{i,l})/(\bar{x}_{i,l+1} - \bar{x}_{i,l}) + \bar{x}_j$. Let $s_l(x_i) = \begin{cases} 1 & \bar{x}_{i,l} \le x_i \le \bar{x}_{i,l+1} \\ 0 & else \end{cases}$, with $\bar{x}_{i,1} = \min(x_i)$; $\bar{x}_{i,L+1} = \max(x_i)$. Then $\hat{x}_j(x_i) = \sum_{l=1}^{L} s_l(x_i)\breve{x}_j(x_i)$ (**Lopes da Silva et al.**, 1989; **Wendling et al.**, 2001; **Ansari-Asl et al.**, 2006).
Bh²D	Bivariate h² directed: $h^2_{i,j} = max_\tau(1 - var(x_j - \hat{x}_j(x_i(t+\tau)))/(var(x_j - \bar{x}_j))))$
Ph²D	Partial h² directed: $h^P_{i,j} = -P_{i,j}/\sqrt{P_{i,i}P_{j,j}}$ with $P = [h^2_{i,j}]^{-1} = [P_{i,j}]$
Bh²U	Bivariate h² undirected: $\bar{h}^2_{i,j} = max\{\lvert h^2_{i,j}\rvert, \lvert h^2_{j,i}\rvert\}$
Ph²U	Partialvariate h² undirected: $\bar{h}^P_{i,j} = -P_{i,j}/\sqrt{P_{i,i}P_{j,j}}$ with $P = [h^2_{i,j}]^{-1} = [P_{i,j}]$
**Mutual Information**	Mutual information in the time domains. Partition amplitude x_i into L bins, which each $x_{i,l}$ has a probability p_l, $l = 1, \ldots, L$. Then Shannon entropy of x is defined as $H(x) = -\sum_{l=1}^{L} p_l \ln p_l$. For a pair of variables $x_i(t)$ and $x_j(t)$, the joint entropy is defined as $H(x_i, x_j) = -\sum_{i,j=1}^{L} p_{i,j} \ln(p_i/p_j)$. (**Paluš et al.**, 2001; **Grassberger et al.**, 1991; **Quiroga et al.**, 2002)
BMITD1	Bivariate mutual information in time domain directed: $I_{i,j} = H(x_i) + H(x_j) - H(x_i, x_j)$
PMITD1	Partial mutual information in time domain directed: $I^P_{i,j} = -P_{i,j}/\sqrt{P_{i,i}P_{j,j}}$ with $P = [I_{i,j}]^{-1} = [P_{i,j}]$
BMITD2	Bivariate Mutual information in time domain directed with estimated errors: $\hat{I}_{i,j} = I_{i,j} + (N(p_{i,j} \neq 0) - N(p_i \neq 0) - N(p_j \neq 0) + 1)/(2N(x_i))$; $N(x_i)$ is the number of x_i.
PMITD2	Partial mutual information in time domain directed with estimated errors: $\hat{I}^P_{i,j} = -P_{i,j}/\sqrt{P_{i,i}P_{j,j}}$ with $P = [\hat{I}_{i,j}]^{-1} = [P_{i,j}]$

BMITU	Bivariate mutual information in time domain undirected: $\bar{I}_{i,j} = \max\{\lvert I_{i,j}\rvert, \lvert I_{j,i}\rvert\}$
PMITU	Partial mutual information in time domain undirected: $\bar{I}^P_{i,j} = -P_{i,j}/\sqrt{P_{i,i}P_{j,j}}$ with $P = [\bar{I}_{i,j}]^{-1} = [P_{i,j}]$
**Coherence**	Linear relationship between paris of channels on frequency domains (**Hinich and Clay**, 1968; **Gotman**, 1983; **Grinsted et al.**, 2004).
BCohF	Bivariate coherence by Fourier transforms: $c_{i,j}(f) = \lvert\langle X_i(f), X^*_j(f)\rangle\rvert^2/\lvert\langle X_i(f)\rangle\rvert\lvert\langle X_j(f)\rangle\rvert$ with $X(f)$ is the Fourier. transforms of x.
PCohF	Partial coherence by Fourier transforms: $c^P_{i,j} = -P_{i,j}/\sqrt{P_{i,i}P_{j,j}}$ with $P = [c_{i,j}]^{-1} = [P_{i,j}]$.
BCohW	Bivariate coherence by Wavelet transforms: $\hat{c}_{i,j}(f) = \lvert\langle \hat{X}_i(f), \hat{X}^*_j(f)\rangle\rvert^2/\lvert\langle \hat{X}_i(f)\rangle\rvert\lvert\langle \hat{X}_j(f)\rangle\rvert$ with $\hat{X}(f)$ is the wavelet. transforms of x.
PCohW	Partial coherence by wavelet transforms: $\hat{c}^P_{i,j} = -P_{i,j}/\sqrt{P_{i,i}P_{j,j}}$ with $P = [\hat{c}_{i,j}]^{-1} = [P_{i,j}]$.
**Granger**	The past values of x_i should contain information that helps predict x_j. Based on the multivariate autogressive models (MVAR) $$X(t) = \sum_{r=1}^{P} A_r X(t-r) + \Xi(t) \quad (1)$$ with $X(t) = [x_1(t), \ldots, x_n(t)]'_{n\times 1}$ and $\Xi(t) = [\xi_1(t), \ldots, \xi_n(t)]'_{n\times 1}$ (**Granger**, 1969; **Seth**, 2010).
GC	Granger causality. a second group of MVAR is necessary: $\tilde{X}(t) = \sum_{r=1}^{P} A_r \tilde{X}(t-r) + \tilde{\Xi}(t)$ with $\tilde{X}(t)$ is $X(t)$ by leaving out the x_j and $\tilde{\Xi}(t) = [\tilde{\xi}_1(t), \ldots, \tilde{\xi}_n(t)]'_{(n-1)\times 1}$. $F_{i,j} = F_{j\to i} = \ln(var(\tilde{\xi}_i)/var(\xi_i))$.
PGC	Partial Granger Causality: $F^P_{i,j} = -P_{i,j}/\sqrt{P_{i,i}P_{j,j}}$ with $P = [F_{i,j}]^{-1} = [P_{i,j}]$
CondGC	Conditional Grange causality (Partial G-Causality in (**Seth**, 2010)). Let $\Sigma(\cdot)$ be a covariance matrix, N be a set of $1, \cdots, n$, and "/" be a removing operator. Then $\Xi_{N/i,j}$ is the vector Ξ without ξ_i, ξ_j and $\Xi_{N/i}$ is the vector Ξ without ξ_i, with $\hat{F}_{i,j} = F_{j\to i\mid N/i,j} = \ln \frac{var(\tilde{\xi}_i) - \Sigma(\tilde{\xi}_i, \tilde{\Xi}_{N/i})\Sigma(\tilde{\Xi}_{N/i}, \tilde{\Xi}_{N/i})^{-1}\Sigma(\tilde{\Xi}_{N/i}, \tilde{\xi}_i)}{var(\xi_i) - \Sigma(\xi_i, \Xi_{N/i,j})\Sigma(\Xi_{N/i,j}, \Xi_{N/i,j})^{-1}\Sigma(\Xi_{N/i,j}, \xi_i)}$.

**Transfer Entropy**	Transfer entropy for Gaussian variables (**???**)
BTED	Bivariate transfer entropy directed: $\mathcal{T}_{i,j} = \max\tau(H(x_i(t+1)\vert X_i(t-\tau)) - H(x_i(t+1)\vert X_i(t-\tau), X_j(t-\tau)) = \log(\vert cov(X_i(t+1))\vert) - \log(\vert cov(X_i(t-\tau))\vert) - \log(\vert cov(X_i(t), X_j(t-\tau))\vert) + \log(\vert cov(X_i(t-\tau), X_j(t-\tau))\vert)$
PTED	Partial transfer entropy directed: $\mathcal{T}^P_{i,j} = -P_{i,j}/\sqrt{P_{i,i}P_{j,j}}$ with $P = [\mathcal{T}_{i,j}]^{-1} = [P_{i,j}]$
BTEU	Bivariate transfer entropy undirected: $\bar{\mathcal{T}}_{i,j} = max\{\vert\mathcal{T}_{i,j}\vert, \vert\mathcal{T}_{i,j}\vert\}$
PTEU	Partial transfer entropy undirected: $\bar{\mathcal{T}}^P_{i,j} = -P_{i,j}/\sqrt{P_{i,i}P_{j,j}}$ with $P = [\bar{\mathcal{T}}_{i,j}]^{-1} = [P_{i,j}]$
$\bar{\mathcal{A}}\mathcal{H}$	From MVAR (1), $\Xi(t) = X(t) - \sum_{r=1}^{p} A_r X(t-r) = \sum_{r=0}^{p} \bar{A}_r X(t-r)$, with $\bar{A}_r = I - A_r$ (**Vidaurre et al.**, 2011).
$\bar{\mathcal{A}}$ Group	
MVAR	$\bar{A}$ in the time domain: $\max_r(\bar{A}_r)$
AS	$\bar{A}$ square: $\sum_r(\bar{A}_r)^2$
Af	$\bar{A}$ in the frequency domain: $\bar{A}(f) = \sum_k \bar{A}(k)exp(i2\pi(k-1)f/Fs)$.
PDC	Partial directed coherence is the normalized Af. PDC ranks the relative interaction strengths with respect to a given signal source. $\pi_{i,j}(f) = \bar{A}_{i,j}(f)/\sqrt{\bar{a}^*_j(f)\bar{a}_j(f)}$, where $\bar{A}(f) = [\bar{A}_{i,j}(f)] = [a_1(f), \cdots, a_n(f)]$. $a_j(f)$ is the jth column of the matrix which means the node j has relation with other nodes. The following normalization properties hold: $0 \le \vert\pi_{i,j}(f)\vert^2 \le 1$; and $\sum_{i=1}^{N} \vert\pi_{i,j}(f)\vert^2 = 1$ for all $0 \le j \le N$ (**Baccalá and Sameshima**, 2001).
PDCF	Partial directed coherence factor (**Baccalá and Sameshima**, 2001): $\hat{\pi}_{i,j}(f) = \bar{A}_{i,j}(f)/\sqrt{\bar{a}^*_j(f)\Sigma^{-1}\bar{a}_j(f)}$
GPDC	Generalized partial directed coherence (**Baccalá**, 2007): $\tilde{\pi}_{i,j}(f) = \bar{A}_{i,j}(f)/(\sigma_i\sqrt{\sum_{k=1}^{N} 1/\sigma^2 \bar{A}_{kj}(f)\bar{A}^*_{kj}(f)})$
$\mathcal{H}$ **Group**	
Hmvar	$\mathcal{H}$_based transfer functions: $H(f) = \bar{A}^{-1}(f)$
DTF	Directed transfer function (**Kaminski and Blinowska**, 1991; **Kaminski et al.**, 2001): $\gamma_{i,j}(f) = \vert H_{i,j}(f)\vert^2/\sum_{j=1}^{N} \vert H_{i,j}(f)\vert^2$.

`ffDTF`	Full frequency directed transfer function (**Kaminski and Liang**, 2005): $\bar{\gamma}_{i,j}(f) = \lvert H_{i,j}(f)\rvert^2 / \sum_f \sum_{j=1}^{N} \lvert H_{i,j}(f)\rvert^2.$
`DC`	Directed coherence (**Baccalá et al.**, 1998): $\hat{\gamma}_{i,j}(f) = \sigma_{jj}\lvert H_{i,j}(f)\rvert^2 / \sqrt{\sum_{j=1}^{N} \sigma_{jj}^2 \lvert H_{i,j}(f)\rvert^2}$
`Smvar`	Power Spectrum $S(f) = X(f)X^*(f) = H(f)\Sigma H^*(f)$
`GGC`	Gewekes Granger Causality (**Geweke**, 1982, 1984): $\tilde{\gamma}_{i,j}(f) = (\sigma_{jj} - \sigma_{ij}^2/\sigma_{jj})\lvert H_{i,j}(f)\rvert^2 / S_{jj}(f)$
`COH1`	Ordinary coherence 1: $C_{i,j} = \lvert S_{i,j}(f)\rvert^2 / (S_{i,i}(f)S_{j,j}(f))$
`COH2`	Ordinary coherence 2: $\hat{C}_{i,j} = \lvert \hat{S}_{i,j}(f)\rvert^2 / (\hat{S}_{i,i}(f)\hat{S}_{j,j}(f))$ with $\hat{S}(f) = H(f)H^*(f)$
`PCOH1`	Partial ordinary coherence 1: $G_{i,j} = \lvert M_{i,j}(f)\rvert^2 / (M_{i,i}(f)M_{j,j}(f))$ with $M(f) = \bar{A}(f)\Sigma^{-1}\bar{A}^*(f)$
`PCOH2`	Partial ordinary coherence 2: $\hat{G}_{i,j} = \lvert \hat{M}_{i,j}(f)\rvert^2 / (\hat{M}_{i,i}(f)\hat{M}_{j,j}(f))$ with $\hat{M}(f) = \bar{A}(f)\bar{A}^*(f)$
`dDTF`	Direct directed transfer function (**Korzeniewska et al.**, 2003): $\breve{\gamma}_{i,j}^2(f) = \bar{\gamma}_{i,j}^2(f)\hat{G}_{i,j}^2(f)$ which combines the information from ffDTF and PCOH2.

Supplementary Table 2. List of parameters used for calculating the connection matrices

	NMM	fMRI	linear	Rössler	Hénon	in Smith et al, 2011
size of windows	800	512	800	800	800	200
	6.4 s	1024 s			600s	
Number of windows	29	19	36	36	19	50
Length of signals	12000	5120	15000	15000	7900	
	96 s	10240 s				
Minimal frequency	1	0.004	1	1	1	0.0027(0.032 for 19,20)
Maximal frequency	20	0.08	90	20	90	0.0533(0.64 for 19,20)
Step of frequency	1	0.004	1	1	1	0.0027(0.032 for 19,20)
Maximum lags	12	12	15	12	12	12
Model orders	5	5	5	10	5	5
Sample frequency	125	0.5	100	100	100	$1/3$

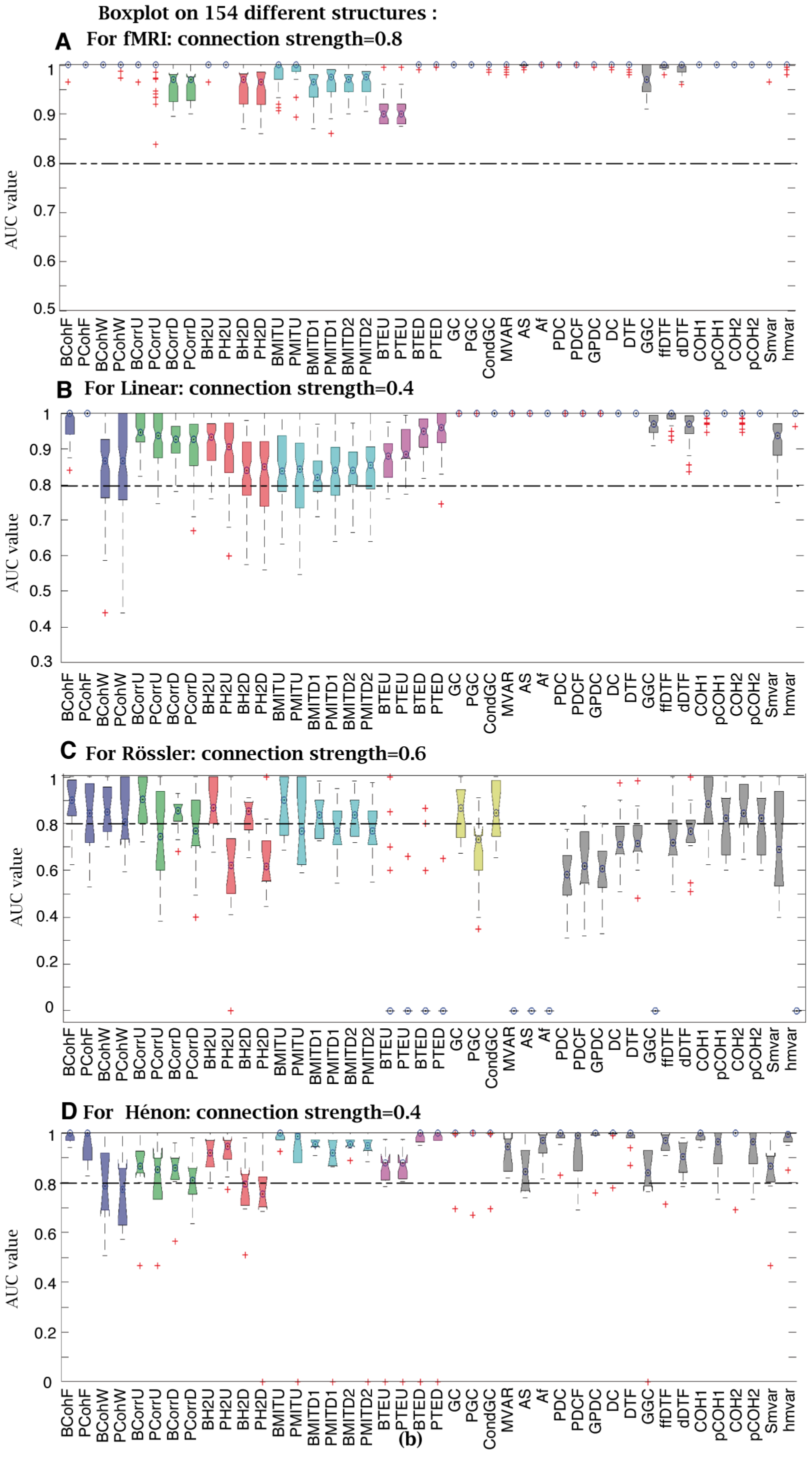

Supplementary Figure 3. Distribution of the AUC values for different structures for the 42 methods. (A) For fMRI, the connection strength was set to 0.8. (B) For linear system, the connection strength was set to 0.4. (C) For Rössler, the connection strength was set to 0.6. (D) For Hénon system, the connection strength was set to 0.4.

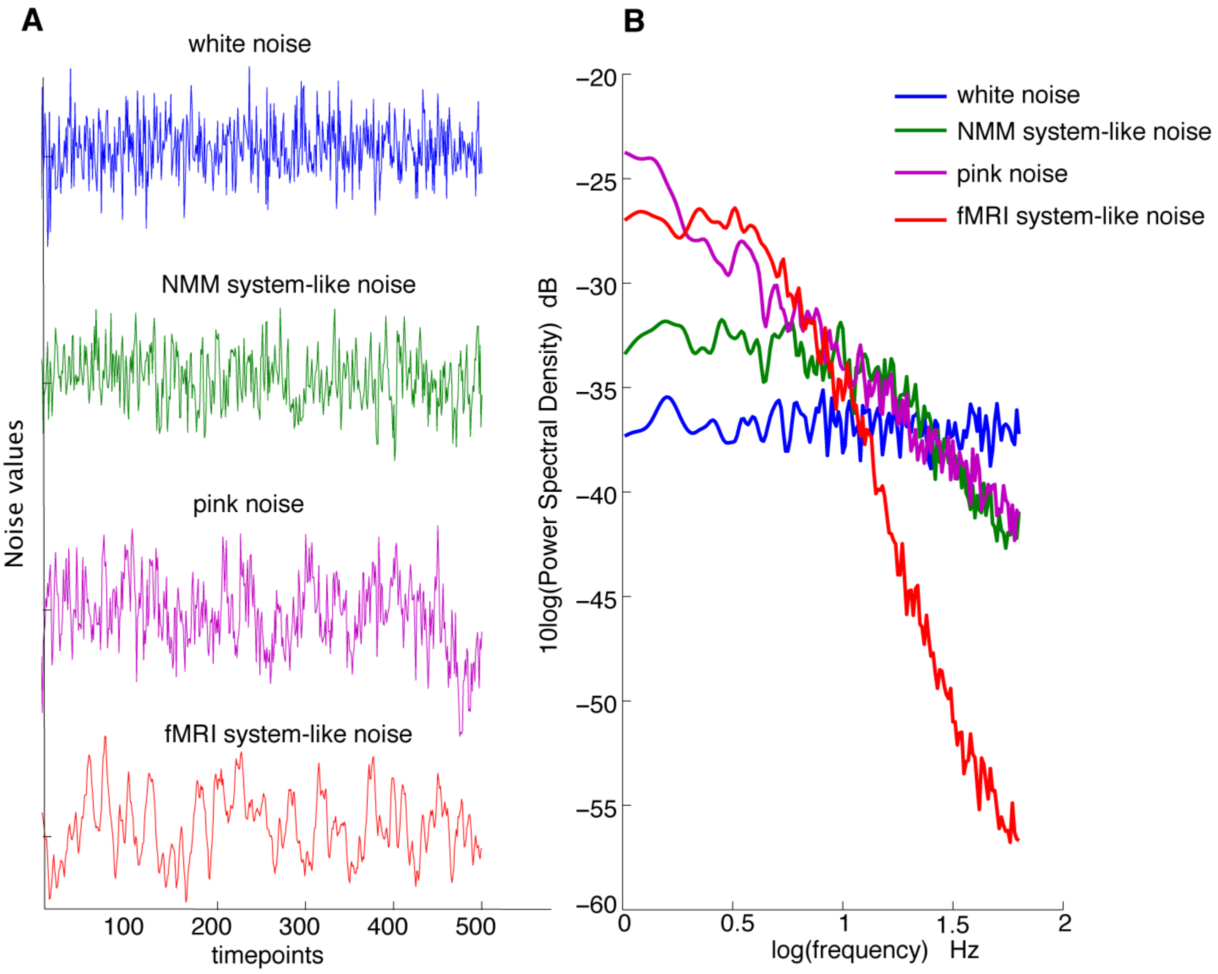

Supplementary Figure 4. Four types of observation noise: white noise, NMM system-like noise, pink noise and fMRI system-like noise. (A) Examples of noise time series. (B) Frequency spectrum for the four types of noise.

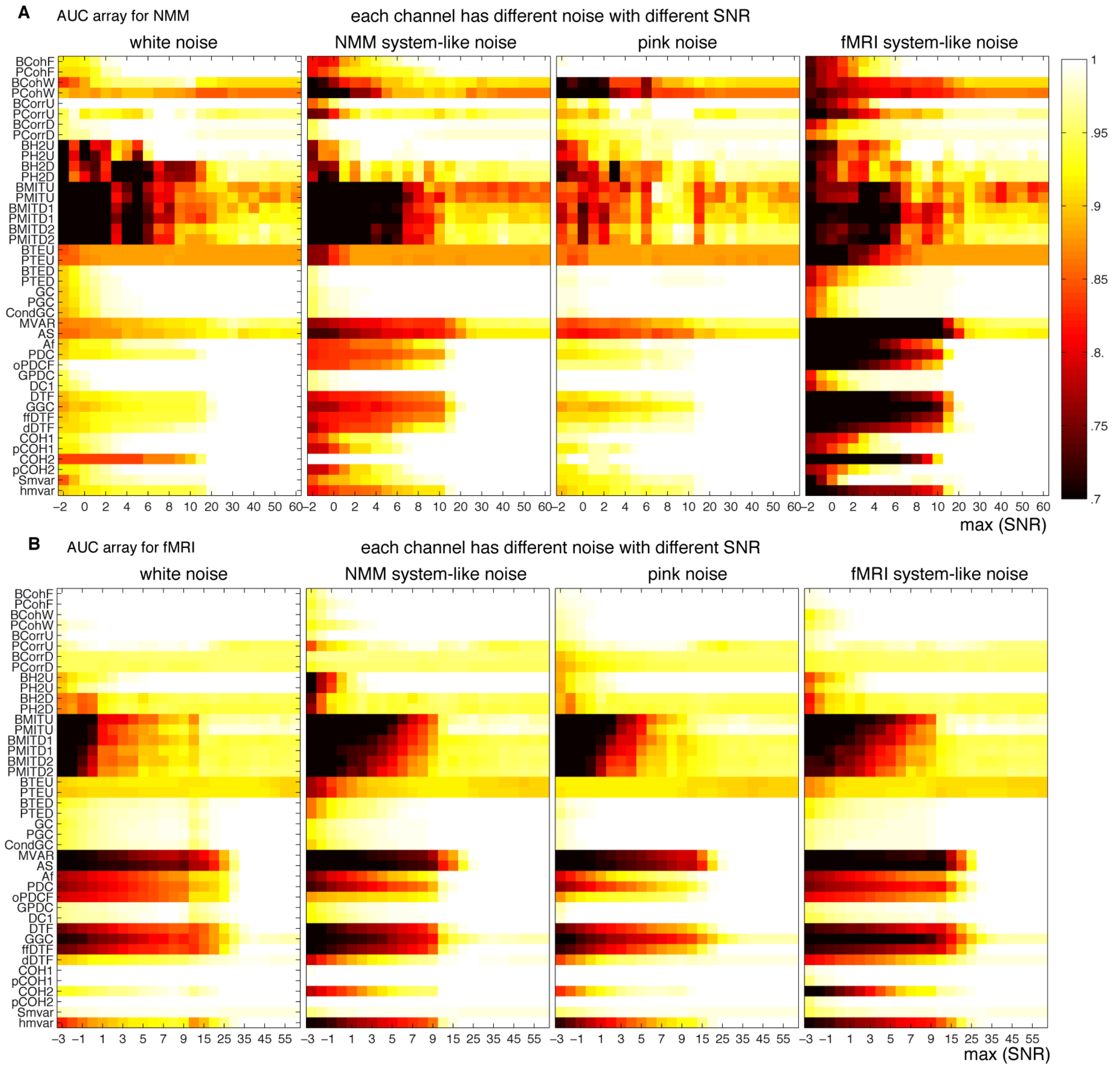

Supplementary Figure 5. Robustness of the 42 methods against the observation noise when each channel receives a different noise with a different SNR (signal-to-noise ratio). The figure shows averaged AUC arrays of the 42 methods as a function of the maximal SNR, (A) for NMM and (B) for fMRI.

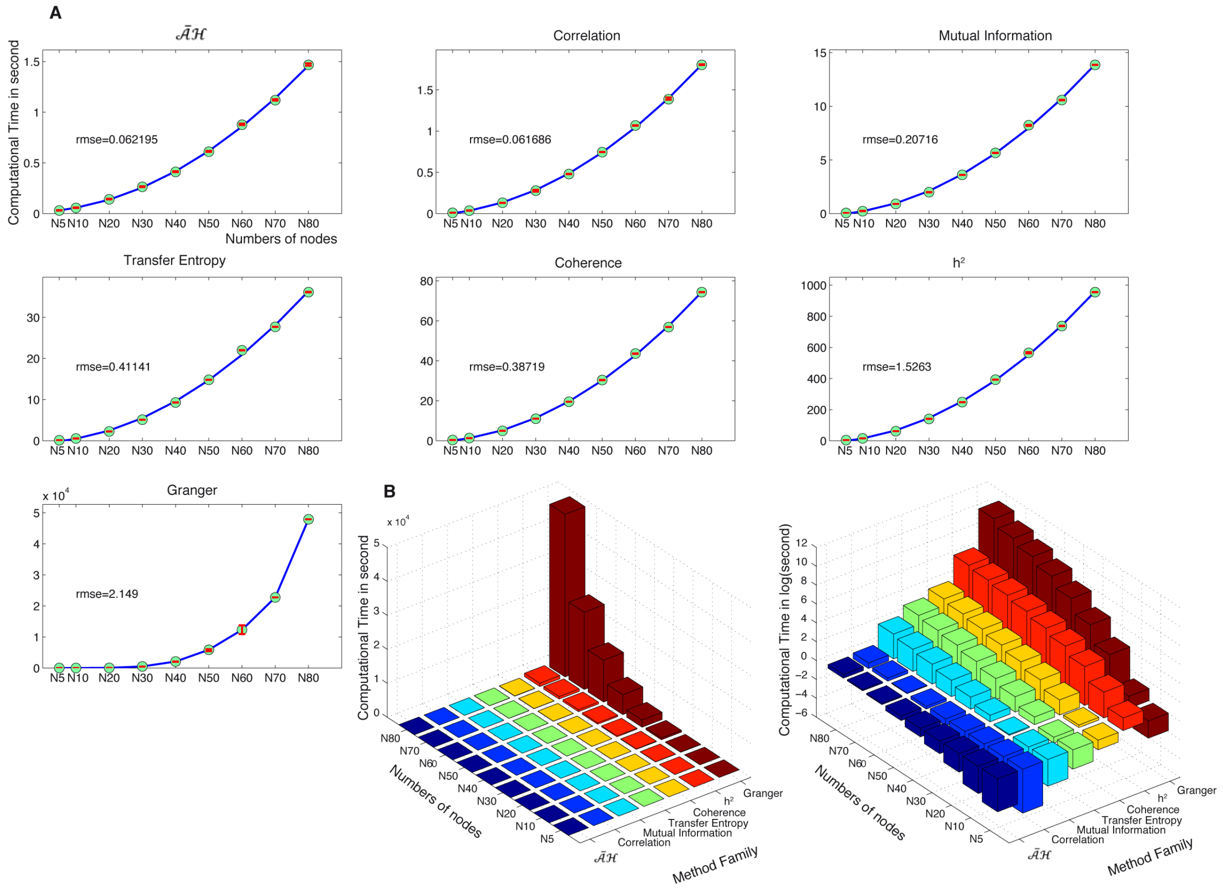

Supplementary Figure 6. (A) The computational time (in seconds) as a function of the number of nodes for the various method families. (B) Computation time in seconds and log(second) as a function of the numbers of nodes and method families.

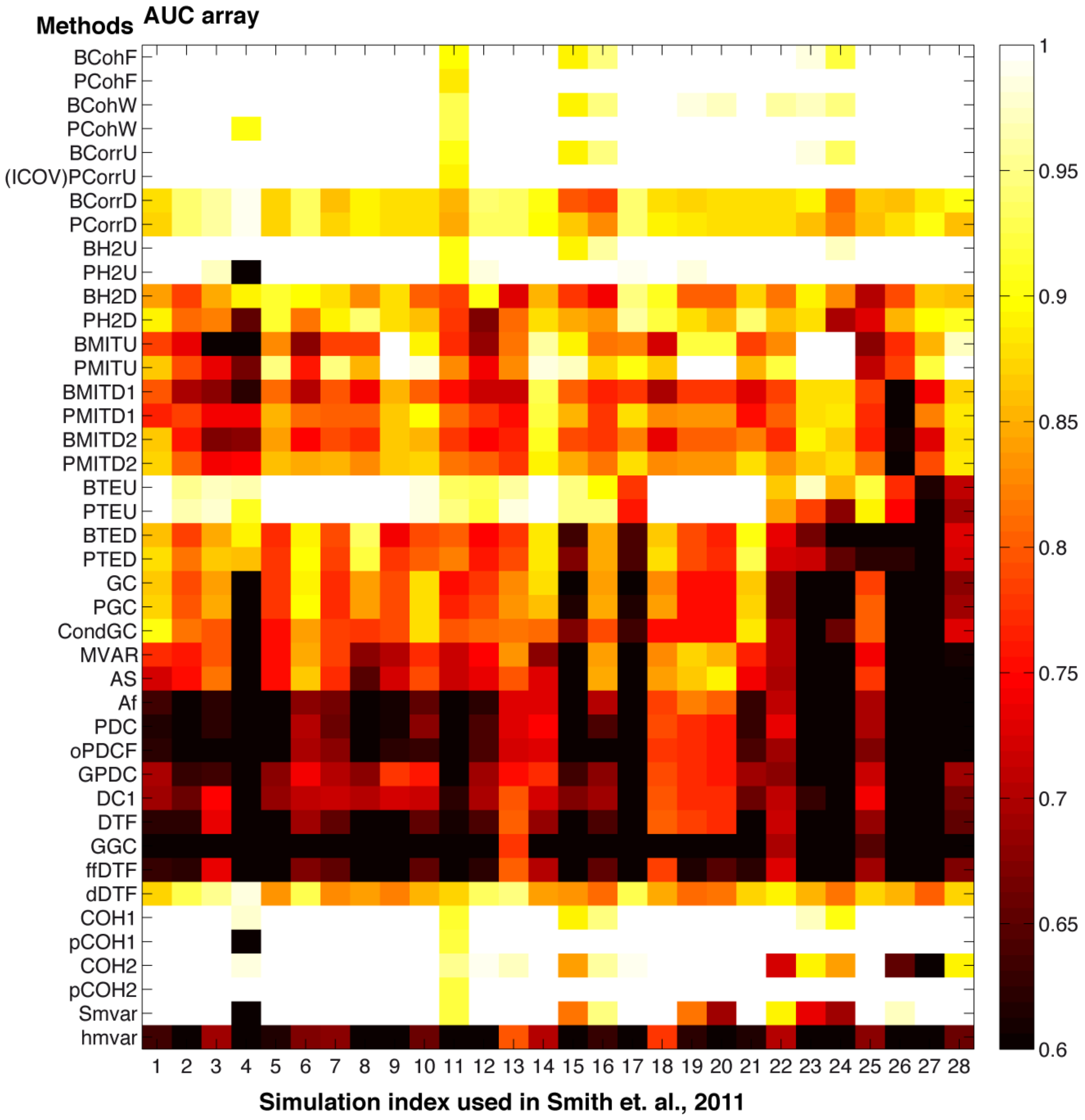

Supplementary Figure 7. Average AUC arrays of 42 methods over different structures as a function of different simulation datasets.

REFERENCES

Ansari-Asl, K., Senhadji, L., Bellanger, J. J., and Wendling, F. (2006), Quantitative evaluation of linear and nonlinear methods characterizing interdependencies between brain signals, *Physical Review E*, 74, 3, 31916

Baccalá, L. (2007), Generalized partial directed coherence, in Digital Signal Processing, 2007 15th International Conference on (IEEE, Baccald, LA), 163–166

Baccalá, L. and Sameshima, K. (2001), Partial directed coherence: a new concept in neural structure determination, *Biological cybernetics*, 84, 6, 463–474

Baccalá, L., Sameshima, K., Ballester, G., Do Valle, A. C., and Timo-loria, C. (1998), Studying the interaction between brain structures via directed coherence and Granger causality, *Applied Signal Processing*, 5, 40–48

David, O., Kiebel, S. J., Harrison, L. M., Mattout, J., Kilner, J. M., and Friston, K. J. (2006), Dynamic causal modeling of evoked responses in EEG and MEG., *NeuroImage*, 30, 4, 1255–72, doi:10.1016/j.neuroimage.2005.10.045

Friston, K. J., Harrison, L., and Penny, W. (2003), Dynamic causal modelling, *Neuroimage*, 19, 4, 1273–1302

Geweke, J. F. (1982), Measurement of linear dependence and feedback between multiple time series, *Journal of the American Statistical Association*, 77, 378, 304–313

Geweke, J. F. (1984), Measures of conditional linear dependence and feedback between time series, *Journal of the American Statistical Association*, 79, 388, 907–915

Gotman, J. (1983), Measurement of small time differences between EEG channels: method and application to epileptic seizure propagation, *Electroencephalography and clinical neurophysiology*, 56, 5, 501–514

Granger, C. W. J. (1969), Investigating causal relations by econometric models and cross-spectral methods, *Econometrica: Journal of the Econometric Society*, 424–438

Grassberger, P., Schreiber, T., and Schaffrath, C. (1991), Nonlinear time sequence analysis, *International Journal of Bifurcation and Chaos*, 1, 03, 521–547

Grinsted, A., Moore, J. C., and Jevrejeva, S. (2004), Application of the cross wavelet transform and wavelet coherence to geophysical time series, *Nonlinear processes in geophysics*, 11, 5/6, 561–566

Hénon, M. (1976), A two-dimensional mapping with a strange attractor, *Communications in Mathematical Physics*, 50, 1, 69–77

Hinich, M. J. and Clay, C. S. (1968), The application of the discrete Fourier transform in the estimation of power spectra, coherence, and bispectra of geophysical data, *Reviews of Geophysics*, 6, 3, 347–363

Jovanović, A., Perović, A., and Borovčanin, M. (2013), Brain connectivity measures: computation and comparison, *EPJ Nonlinear Biomedical Physics*, 1, 1, 2, doi:10.1186/epjnbp2

Kaminski, M., Ding, M., Truccolo, W. A., and Bressler, S. L. (2001), Evaluating causal relations in neural systems: Granger causality, directed transfer function and statistical assessment of significance, *Biological cybernetics*, 85, 2, 145–157

Kaminski, M. and Liang, H. (2005), Causal influence: advances in neurosignal analysis., *Critical reviews in biomedical engineering*, 33, 4, 347

Kaminski, M. J. and Blinowska, K. J. (1991), A new method of the description of the information flow in the brain structures, *Biological cybernetics*, 65, 3, 203–210

Korzeniewska, A., Maczak, M., Kamiski, M., Blinowska, K. J., and Kasicki, S. (2003), Determination of information flow direction among brain structures by a modified directed transfer function (dDTF) method, *Journal of neuroscience methods*, 125, 1, 195–207

Lopes da Silva, F., Pijn, J. P., and Boeijinga, P. (1989), Interdependence of EEG signals: linear vs. nonlinear associations and the significance of time delays and phase shifts., *Brain topography*, 2, 1-2, 9–18

Moran, R., Pinotsis, D., and Friston, K. (2013), Neural masses and fields in dynamic causal modeling., *Frontiers in computational neuroscience*, 7, May, 57, doi:10.3389/fncom.2013.00057

Paluš, M., Komárek, V., Hrnčí, Z., and Štěrbová, K. (2001), Synchronization as adjustment of information rates: detection from bivariate time series, *Physical Review E*, 63, 4, 46211

Quiroga, R. Q., Kraskov, A., Kreuz, T., and Grassberger, P. (2002), Performance of different synchronization measures in real data: a case study on electroencephalographic signals, *Physical Review E*, 65, 4, 41903

Rössler, O. E. (1979), Continuous chaos: four prototype equations, *Annals of the New York Academy of Sciences*, 316, 376–392

Seth, A. K. (2010), A MATLAB toolbox for Granger causal connectivity analysis., *Journal of neuroscience methods*, 186, 2, 262–73, doi:10.1016/j.jneumeth.2009.11.020

Vidaurre, C., Sander, T. H., and Schlögl, A. (2011), BioSig: the free and open source software library for biomedical signal processing., *Computational intelligence and neuroscience*, 2011, 935364, doi:10.1155/2011/935364

Wendling, F., Bartolomei, F., Bellanger, J. J., and Chauvel, P. (2001), Interpretation of interdependencies in epileptic signals using a macroscopic physiological model of the EEG, *Clinical Neurophysiology*, 112, 7, 1201–1218
