# Supplementary material for: A systematic framework for functional connectivity measures
Source: Front Neurosci. 2014 Dec 9;8:405. doi: 10.3389/fnins.2014.00405 (PMC4260483; doi:10.3389/fnins.2014.00405)
Supplement: Supplementary file 2 [file Presentation2.PDF]

User Guide

MULTIPLE CONNECTIVITY ANALYSIS (MULAN)
A MATLAB TOOLBOX

MULAN 1: EVALUATION

Huifang E Wang, Christian G Bénar, Pascale P Quilichini, Karl J Friston, Viktor Jirsa and Christophe Bernard

Reference paper: **A systematic framework for functional connectivity measures**, submitted to Frontiers in NEUROSCIENCE

Institut de Neurosciences des Systèmes, UMR INSERM 1106, Aix-Marseille Université, 13005 Marseille, France
The Wellcome Trust Centre for Neuroimaging, University College London, Queen Square, London, UK

Contents

1. Introduction 3
1.1. MULAN 1 Environment 3
1.2. MULAN setup 3
2. MULAN interface 4
2.1. Simulated data 5
2.2. Setting Panels 6
2.3. Methods list 7
2.4. Calculation of connection matrices 8
2.5. Demonstration of the results 9
2.6. Demonstration of average results 10
2.7. Detailed analyses 11
2.8. Evaluation of methods 12
3. MULAN cluster computing 13
3.1. Calculation 13
3.2. Demonstration 14
Appendix A. MULAN data structures 15
Appendix B. Preprocessing MULAN data 16
B.1. Time frequency analysis 16
B.2. Signal processing 17
B.3. Signal distribution 17
Acknowledgements 18

1. Introduction

The MULAN (MULtiple connectivity ANalysis) MATLAB toolbox helps researchers to evaluate connectivity analysis methods in a systematic way. Prior to applying specific methods to a given dataset, MULAN can be used to generate relevant simulated signals, identify valid parameter ranges for the methods, and evaluate their performance and robustness against underlying connection strengths, graph structures and noise levels. Lastly, new methods can easily be added and tested using this toolbox.

This user guide describes the usage of the MULAN interface as well as using a compute cluster to perform the computations.

1.1. **MULAN 1 Environment.**

MULAN 1 has been tested on three versions of Matlab: MATLAB 7.10.0.499 (R2010a) 64-bit (maci64) for Mac OS X, MATLAB 7.13.0.564 (R2011b) 64-bit (glnxa64) for Linux, MATLAB 7.10.0.499 (R2010a) 64-bit (win64) for Windows.

1.2. **MULAN setup.**

To begin using MULAN, just place the MULAN1 files in any accessible folder on your computer and make sure to either set the MULAN1 folder as the current path in MATLAB or add the folder containing MULAN1 to the MATLAB path.

2. MULAN INTERFACE

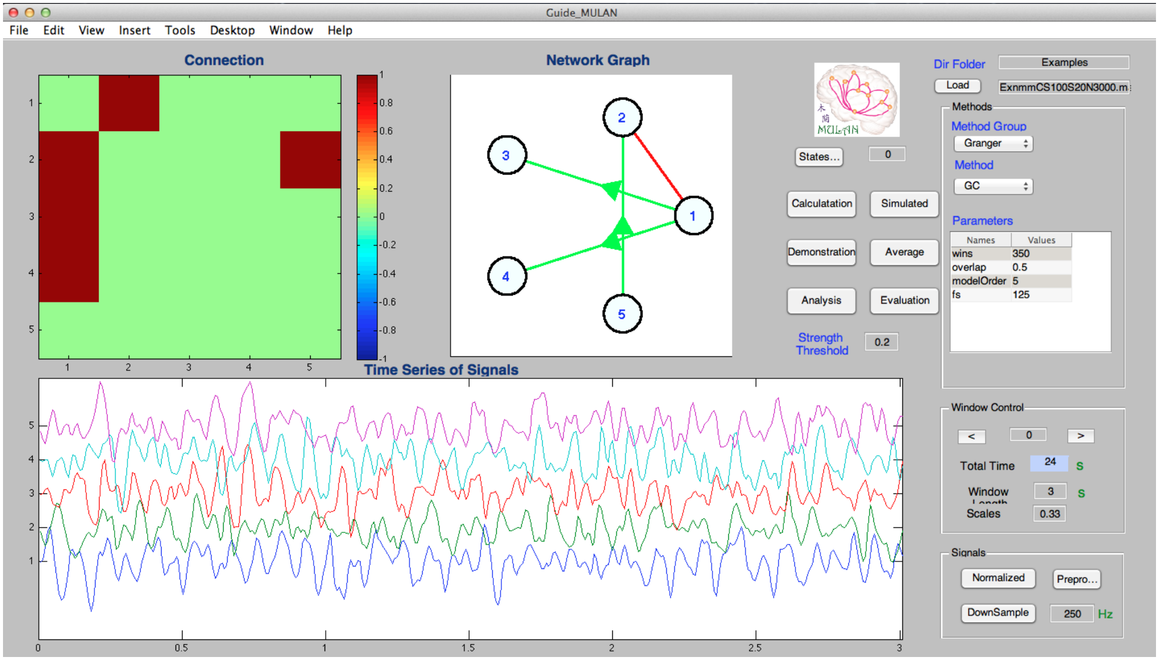

Figure 1 | The interactive graphic user interface (GUI) of MULAN1. In this GUI, the top panels are the connection matrix and network graph, which are the results from given methods and parameters set by the Methods panel. The time series of signals of the given dataset (bottom) can be set by the window control panel.

Start MULAN by two simple options.

Option 1: : Just type "Guide_MULAN" at the Matlab command line and hit enter.

Option 2: : Open file Guide_MULAN.m in MULAN folder, then click the button RUN.

2.1. Simulated data.

If we want to generate the simulated data, please click Simulated button.

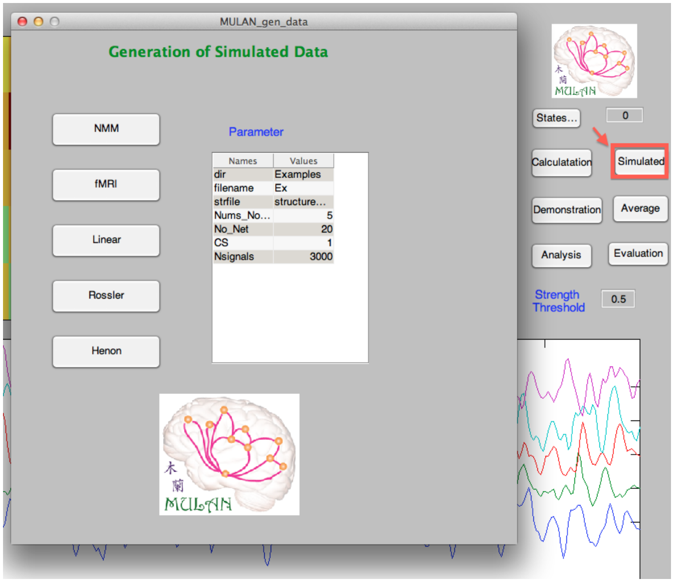

Figure 2 | Generation of Simulated Data.

The parameters which we can choose are:

dir : the folder which are used to store the results.

filename : the base name you choose; the software will add more details on the filenames of each dataset. For example, 'nmm' for model type, CS for connection strength.

strfile : the structure file which stores the information about underlying structures.

Nums_Node : The numbers of nodes for the datasets

CS : The connection strengths

Nsignals : The lengths of signals which you would like to generate.

Then choose the types of models listed on the left, for example, click NMM button to generate NMM datasets. There is the message dialog to inform you if it has finish successfully or not.

2.2. Setting Panels.

First we need to specify the following terms, ref to Fig. 3.

Dir Folder : We put the folder name that has the data file we are about to analyze.

File name : We put the file name in which data stores. Put the name of data file and then click Load button.

Method Group : We need choose the method family which we want to use.

Method : We can specify a method from a specified family. We use Granger family as an exmaple in Fig. 3. A methods list on the right provides the abrreviation and corresponding notations about the methods.

Parameters : Once we've chosen the method family, the parameters which need to be specified will be listed in a table.

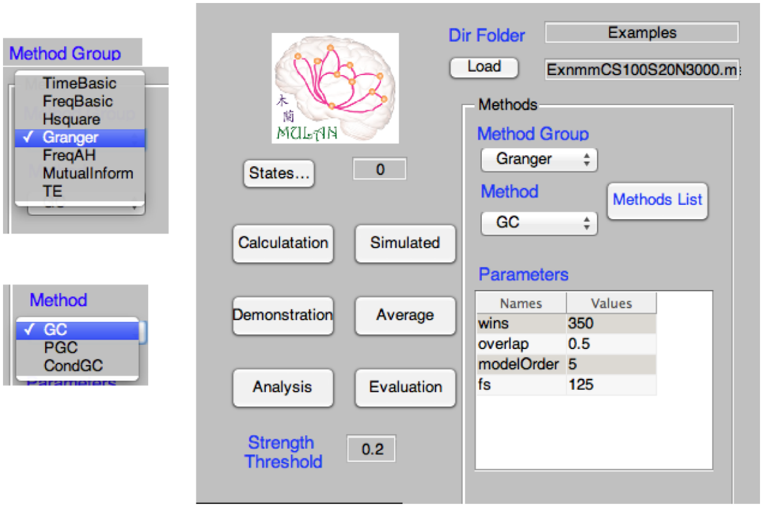

Figure 3 | setting Panels

2.3. Methods list.

Table 1 | Abbreviation and notations of the connectivity analysis methods. Please refer to the submitted paper for the mathematical definitions.

Abbr.	Notations	Abbr.	Notations
	Correlation		
BCorrU	Bivariate correlation undirected	PCorrU	Partial correlation undirected
BCorrD	Bivariate correlation directed	PCorrD	Partial correlation directed
	$\mathtt{h}^2$		
$\mathtt{Bh}^2\mathtt{U}$	Bivariate $\mathtt{h}^2$ undirected	$\mathtt{Ph}^2\mathtt{U}$	Partial $\mathtt{h}^2$ undirected
$\mathtt{Bh}^2\mathtt{D}$	Bivariate $\mathtt{h}^2$ directed	$\mathtt{Ph}^2\mathtt{D}$	Partial $\mathtt{h}^2$ directed
	Mutual Information		
BMITU	Bivariate mutual information undirected	PMITU	Partial mutual information undirected
BMITD1	Bivariate mutual information directed 1	PMITD1	Partial mutual information directed 1
BMITD2	Bivariate mutual information directed 2	PMITD2	Partial mutual information directed 2
	Coherence		
BCohF	Bivariate Fourier transforms	PCohF	Partial Fourier transforms
BCohW	Bivariate wavelet transforms	PCohW	Partial wavelet transforms
	Granger family		
GC	Granger causality	PGC	Partial Granger causality
CondGC	Conditional Granger causality		
	Transfer Entropy		
BTEU	Bivariate transfer entropy undirected	PTEU	Partial transfer entropy undirected
BTED	Bivariate transfer entropy directed	PTED	Partial transfer entropy directed
	$\bar{\mathcal{A}}\mathcal{H}$		
Af	$\bar{A}$ in the frequency domain	hmvar	$\mathcal{H}$ in the frequency domain
PDC	Partial directed coherence	DTF	Directed transfer function
PDCF	Partial directed coherence factor	DC	Directed coherence
GPDC	Generalized partial directed coherence	ffDTF	Full frequency directed transfer function
GGC	Gewekes Granger Causality	dDTF	Direct directed transfer function
PCOH1	Partial ordinary coherence 1	COH1	Ordinary coherence 1
PCOH2	Partial ordinary coherence 2	COH2	Ordinary coherence 2
MAVR	$\bar{A}$ in the time domain	Smvar	Power Spectrum
AS	$\bar{A}$ square		

2.4. Calculation of connection matrices. (Fig. 4).

(1) After specifying the data file, method and calculation parameters, click the button Calculation.
(2) Once the calculation finishes, the massage dialog will pop up to inform you that the calculation completed.
(3) Then you will find your results in the same folder of the data with a subfolder Results, which will allow you to access your results afterward so that you can save calculation time when the method parameters are the same.

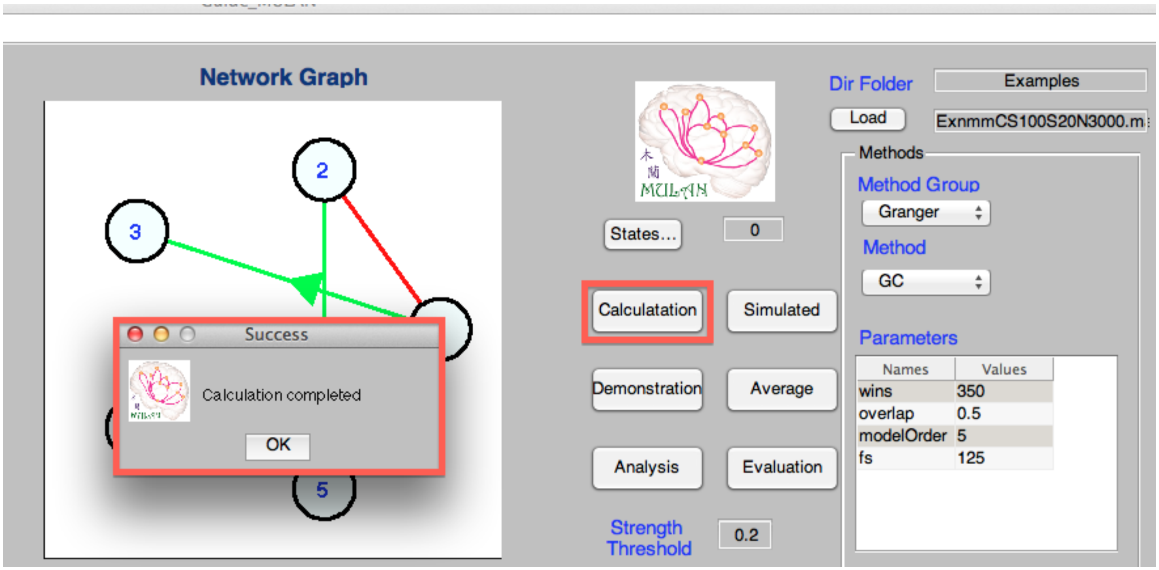

Figure 4 | Calculation of the computed connection matrices by choosing Methods and Parameters

2.5. Demonstration of the results.

After calculation of the computed connection matrices, the results can be visualized (Fig.5):

Step 1 Choose the threshold of connection strength in [0, 1].

Step 2 Click the button Demonstration will show the connection matrix and graph corresponding to the data segment during the current windows.

Step 3 Choose current data segment by directly inputing the current start time point, or by clicking backward/forward buttons.

Step 4 The three windows, Connection, Network Graph and time-series of signals, will change accordingly.

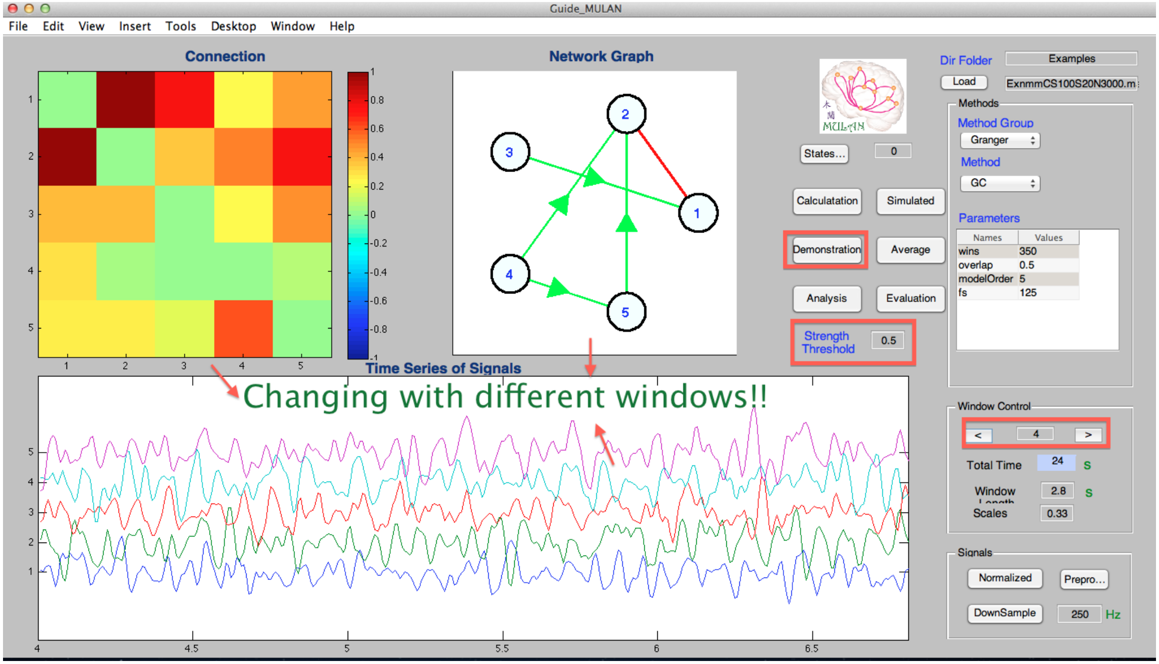

Figure 5 | Demonstration of the results.

2.6. **Demonstration of average results.**

As we suggested in the paper, the average value is more stable than the results from a single window. In order to view average results over the whole dataset, please click the button Average (Fig.6).

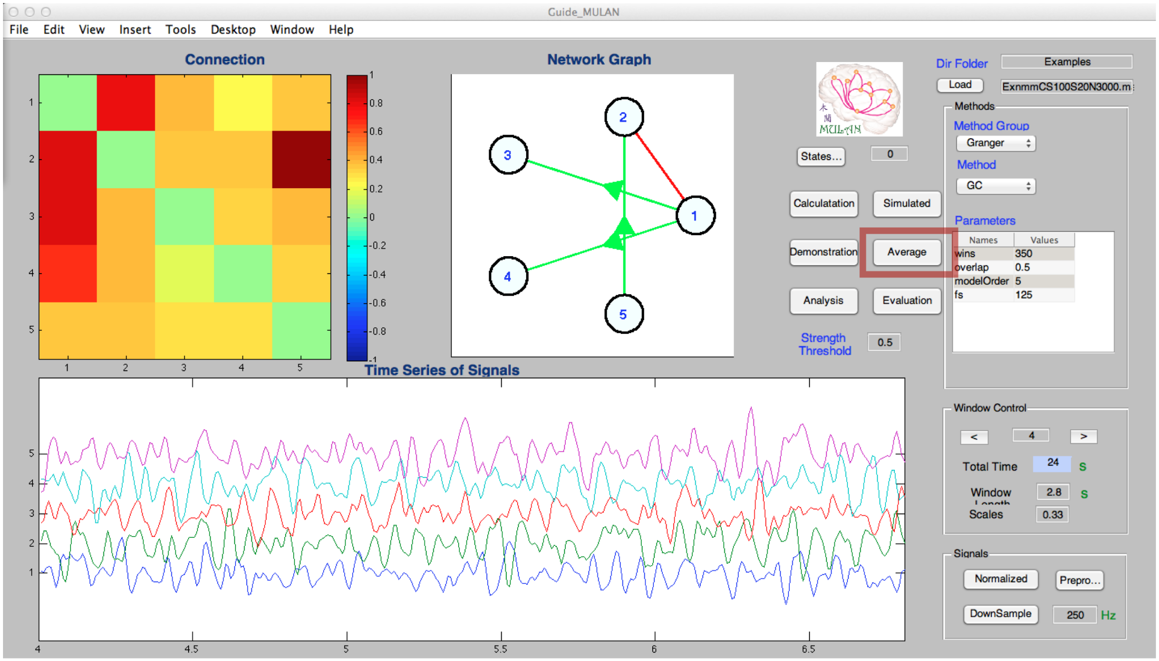

Figure 6 | Demonstration of the results.

2.7. Detailed analyses.

MULAN can provide detailed results by clicking button Analysis. A pop-up figure describes the connectivity strengths between the pairs of channels as function of windows shown in blue and average value over all windows shown in purple, as shown in Fig. 7.

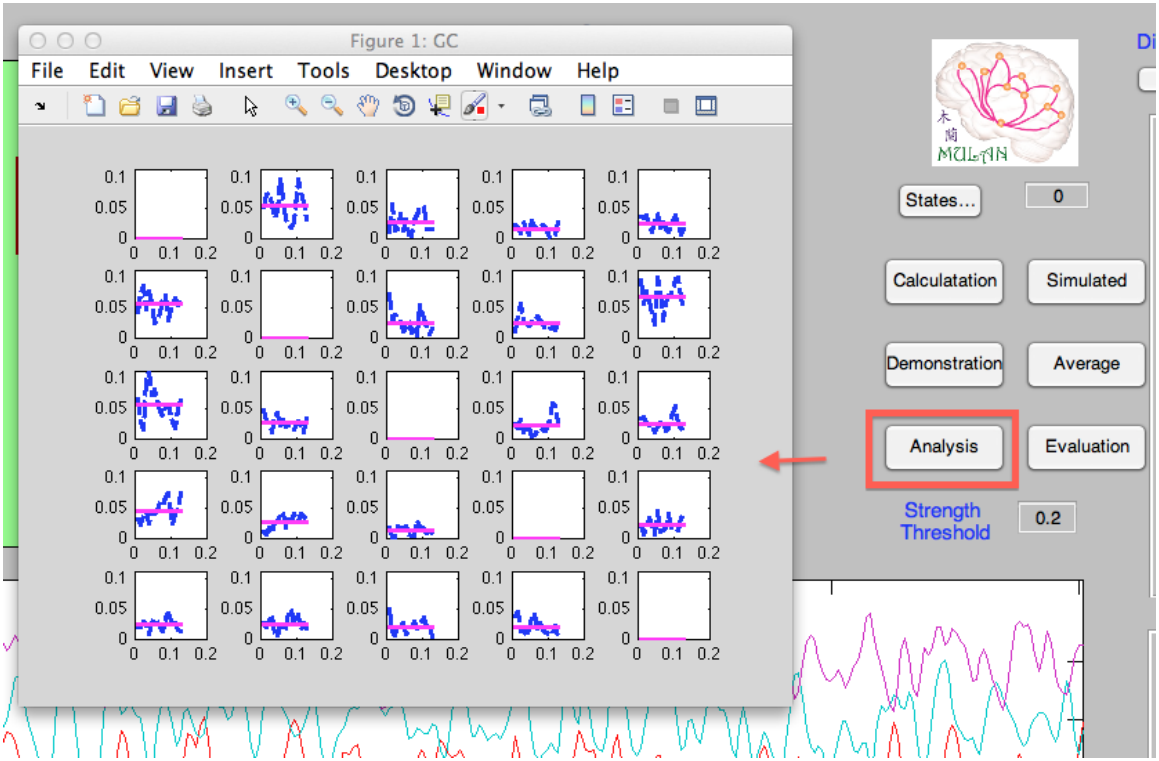

Figure 7 | Detail results on all windows.

2.8. Evaluation of methods.

Click the button Evaluation to bring up a figure displaying evaluation results of methods. Note that the Evaluation button only works in the cases of known ground truth structures. Please refer to Fig. 8.

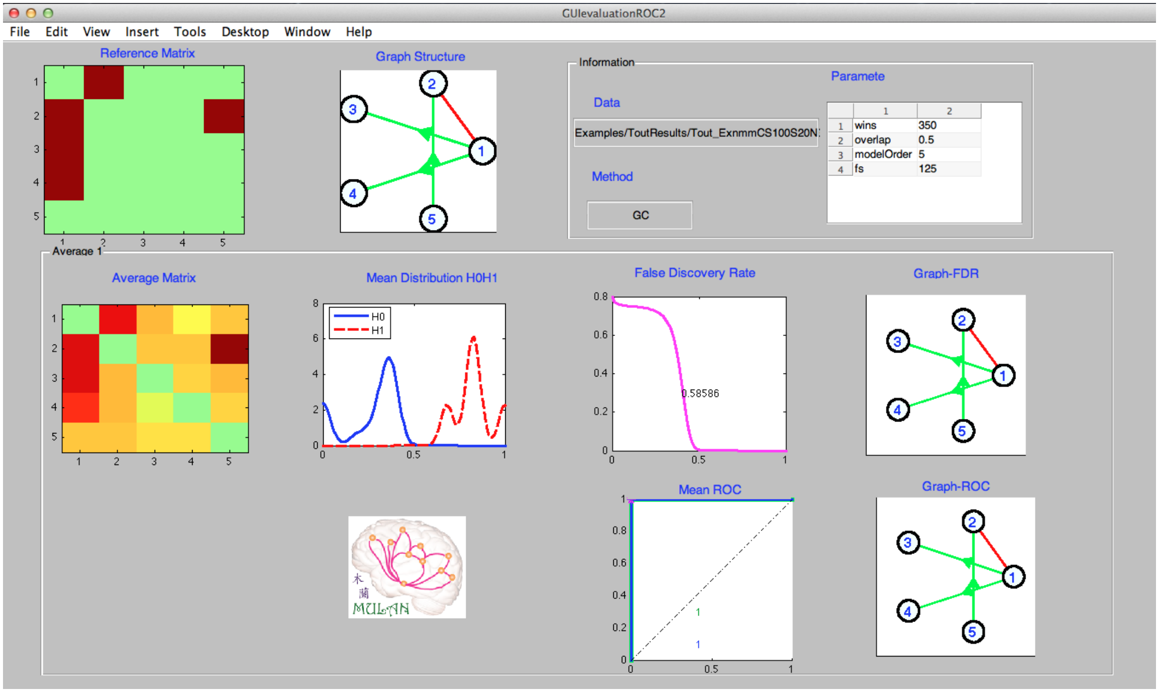

Figure 8 | Evaluation of results from Granger causality.

Fig. 8 contains the results about *average matrix*, H_0/H_1 *distributions*, *the false discovery rate (FDR)* and *the receiver operating characteristic (ROC)*. Two graphs, to the right of FDR and ROC, are shown by choosing thresholds from two ways: 1) From FDR where FDR=0, 2) From ROC where the optimal threshold corresponds to the point closest to the upper left corner.

3. MULAN CLUSTER COMPUTING

3.1. Calculation.

This section helps users to test their connectivity analysis methods or results systematically, e.g. when applying all methods with different parameters on the different datasets in batch.

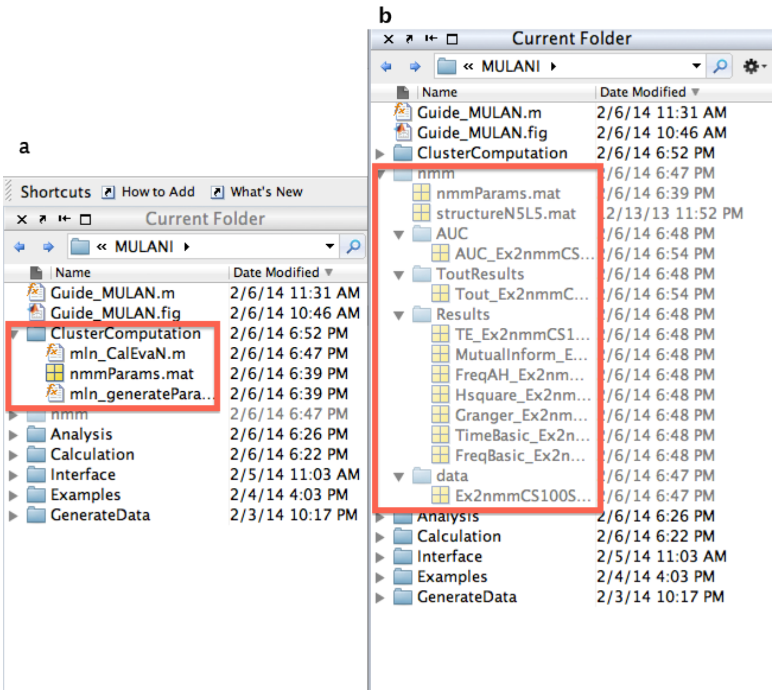

Figure 9 | Evaluation of results from Granger causality.

A demo function is called $mln\_CalEvaN.m$ which you can find it in the folder $'MULAN1/ClusterComputation/'$, shown as in Fig.9(a). This function can generate the datasets, calculate the connection strengths by using all given connectivity analysis methods and calucate the AUC (area under curve) value of ROC (Receiver operating characteristic).

The result files from this function will be found in a given folder name which has four subfolders: 1) $/data$ for datasets 2) $/Results$ for the different methods, 3) $/ToutResults$ for all results in one file and 4) $/AUC$ for the AUC results , as shown in Fig.9(b).

3.2. **Demonstration.**

A demo function is called *mln_showAUC.m* which you can find it in the folder $'MULAN1/Evaluation/'$. This demo function will produce Fig.10 as follows.

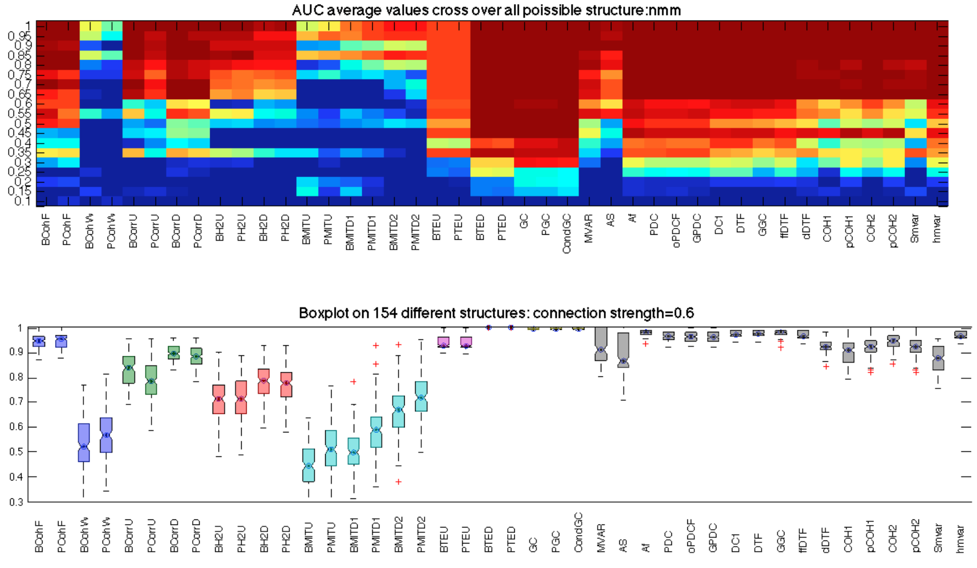

Figure 10 | Results from *mln_showAUC.m*

This demo function shows how to produce AUC average arrays and the boxplot on the different structures in the figures of the submitted paper.

Appendix A. MULAN data structures

This section describes how to set the data structures of the datasets.
Please refer to the structures of the example dataset as an example:
'/MULAN1/Examples/data/ExnmmCS100S20N3000.mat'. Data structures:

- lfp(Obligation): $nchannel \times timeseries$ double. $nchannel$ is the number of signal channels.
- Params(Obligation): structure
  - fs (Obligation): sample frequency
  - str(Option): $1 \times nchannel$ list of the names of the channels. If this field does not exist or is empty, the names will be numbered.
- Connectivity(Option): $nchannel \times nchannel$ double $M_{i,j}$. The value $M_{i,j}$ is the connection strength from channel j to channel i.

Note that the structure "Params" can include many fields but "fs" is only mandatory field for MULAN Toolbox. The variable 'Connectivity' is only necessary for simulated datasets.

Appendix B. Preprocessing MULAN data

MULAN Toolbox provides some features to process the data.

B.1. Time frequency analysis.

(1) Click button Preprocess in MULAN main GUI on Fig. 11.
(2) A parameter setting interface pops up. It allows you to change parameters to spectrum analysis. Then you can choose buttons Fourier or button Wavelet to transform. (Fig. 11).
(3) Then a figure including the spectrum subfigures with each channel will pop out.

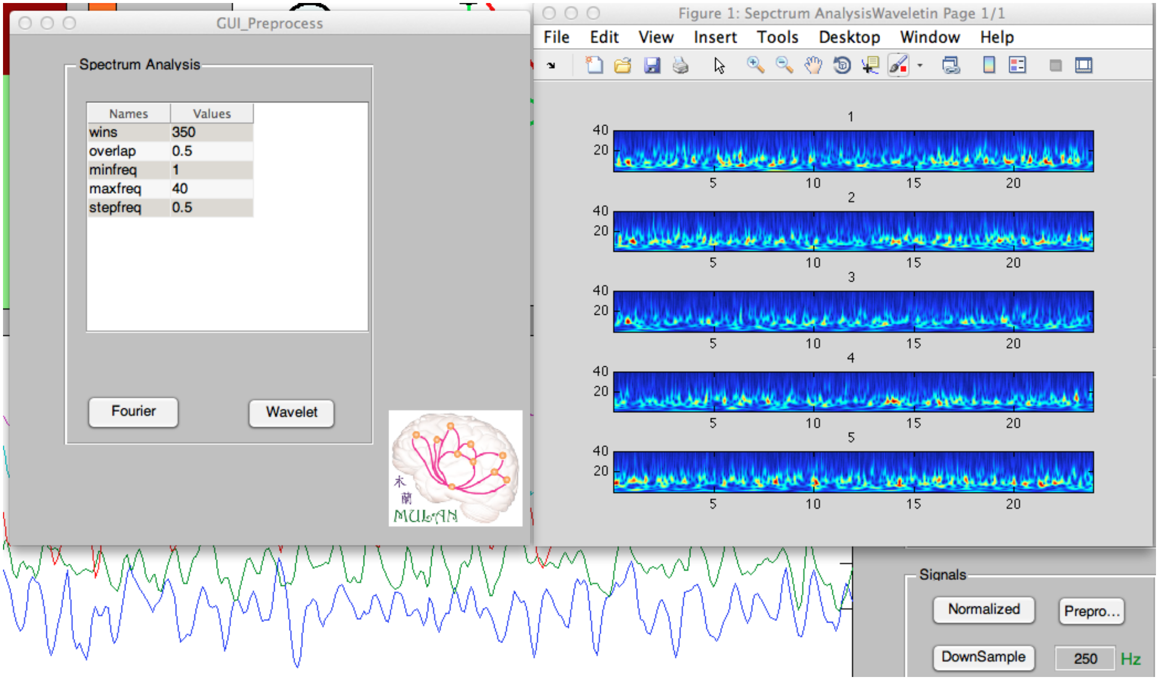

Figure 11 | The time frequency analysis

B.2. **Signal processing.**

(1) Click the button Normalized in the "Signals" panel MULAN main GUI on Fig. 11. Then the normalized data will show up. If we want to go back to the original data, please click the button Load.

(2) If you want to down sample the data, please first choose the expected frequency x Hz, then click the button DownSample. Note that x must be less than the original sample frequency.

(3) Then a new file named *"...fsx.mat"* will be created and saved in the current folder.

B.3. **Signal distribution.** See Fig. 12.

Click the button States Plot in MULAN main GUI on Fig. 11 after setting the value of delay τ next to this button. This will display a figure with subfigures, each of them describing the signal distribution between channel i and channel j with delay τ.

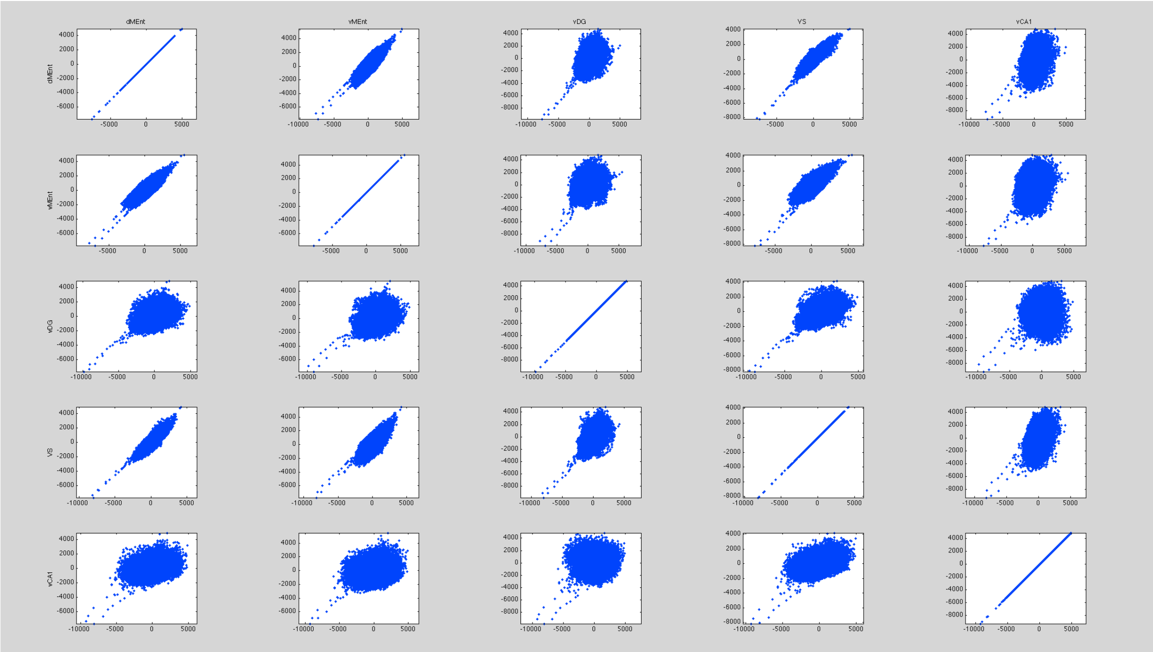

Figure 12 | Signal distribution between channels with a given time delay

Acknowledgements

Special thanks to Marmaduke Woodman for software testing and English editing of this user guide.

We are very thankful to the following open source toolboxes from which we were able to derive functions and methods:

- Statistical Parametric Mapping (SPM), from the wellcom Trust Centre for Neuroimaging, available at `http://www.fil.ion.ucl.ac.uk/spm/`
- Granger Causal Connectivity Anlysis toolbox, from Anil K.Seth, available at `http://www.anilseth.com/`.
- BIOSIG-toolbox, from Alois Schloegl, available at `http://biosig.sf.net/`.
- BSMART: A Matlab/C Toolbox for Analyzing Brain Circuits, from Jie Cui, Lei Xu, Steven L. Bressler, Mingzhou Ding, Hualou Liang, available at `http://www.brain-smart.org/`.
- Neurophysiological Biomarker Toolbox (NBT), from Simon-Shlomo Poil, `http://www.nbtwiki.net/`.
